# A human 3D BBB chip model of acute stroke simulating a reversible penumbra

**DOI:** 10.1371/journal.pone.0352263

**Published:** 2026-07-14

**Authors:** Kyung A Kwak, Eun-Young Shin, Kyung Sik Yi, Chi-Hoon Choi

**Affiliations:** 1 Department of Radiology, College of Medicine, Chungbuk National University, Cheongju, Republic of Korea; 2 Department of Biochemistry, Chungbuk National University College of Medicine and Medical Research Center, Chungbuk National University Hospital, Cheongju, Republic of Korea; 3 Department of Radiology, Chungbuk National University Hospital, Cheongju, Republic of Korea; Eötvös Loránd Research Network Biological Research Centre, HUNGARY

## Abstract

Ischemic stroke is a leading cause of mortality and disability worldwide. However, existing models often fail to replicate key aspects of human pathophysiology, particularly blood–brain barrier (BBB) dysfunction and the salvageable ischemic penumbra. We developed a three-dimensional BBB chip model of acute ischemic stroke that reproduces penumbra-like, partially reversible BBB injury. This platform integrates a microfluidic BBB chip (Emulate) with parallel Transwell inserts to facilitate complementary structural, molecular, and functional analyses. Ischemia-like injury was induced using 2.5 μM antimycin A for 1 hour under oxygen-glucose deprivation conditions, followed by medium replacement to simulate reperfusion. Therapeutic hypothermia (33°C) was also applied during the reperfusion phase. The combination of reperfusion and hypothermia resulted in the most pronounced restoration of BBB integrity compared with reperfusion alone. The Emulate chip enabled structural evaluation of endothelial morphology, while the transwell model showed concordant recovery of BBB-related markers, including ZO-1 and VE-cadherin, along with decreased expression of the hypoxia-associated marker HIF-1α. This integrated platform enabled evaluation of BBB injury and recovery under ischemia- and reperfusion-like conditions. Our human cell-based 3D BBB stroke model captures key BBB-related features of penumbra-like injury and provides a human-relevant in vitro platform for investigating stroke pathophysiology and evaluating therapeutic strategies.

## Introduction

Stroke is a leading cause of mortality worldwide and the leading cause of adult disability, necessitating continued research in neuroscience and medicine. Strokes are broadly classified into hemorrhagic and ischemic types, with ischemic stroke accounting for approximately 80% of all cases [1]. In ischemic stroke, interruption of cerebral blood flow results in oxygen and nutrient deprivation, leading to neuronal injury through multiple pathophysiological mechanisms [2]. Because the brain consumes approximately 20% of the body’s oxygen and 25% of its glucose, it is particularly vulnerable to ischemic injury, which can result in extensive neural damage [3].

Traditionally, investigations of ischemic stroke pathophysiology and therapeutic development have relied on animal models (in vivo) and conventional cell culture systems (in vitro) [4]. The middle cerebral artery occlusion (MCAO) model in rodents is among the most widely used experimental stroke models because it produces relatively reproducible ischemic lesions [5]. However, such models are associated with several limitations, including procedural variability, high mortality, and species-specific physiological differences. In addition, reproducibly modeling a reversible ischemic penumbra often requires large numbers of experimental animals [6,7]. Increasing ethical concerns and regulatory restrictions regarding animal experimentation further highlight the need for alternative human-relevant experimental models [6,8].

Against this backdrop, microphysiological systems (MPS) have emerged as promising platforms for stroke research [9,10]. Three-dimensional blood–brain barrier (3D BBB) chip systems allow more physiologically relevant simulation of human BBB structure and function. Recent advances in human BBB and neurovascular unit (NVU) modeling, including neurovascular unit-on-a-chip and human brain vasculature-on-a-chip systems, have enabled the development of increasingly sophisticated in vitro models that better recapitulate the human brain microenvironment [11,12]. These platforms provide precise control of the cellular microenvironment and support integrated structural and functional analyses, making them attractive tools for preclinical therapeutic evaluation [9,13–16]. Previous studies using MPS-based stroke models have investigated BBB disruption, inflammatory responses, and neuroprotective mechanisms under ischemic conditions [14,16,17].

Therapeutic hypothermia (TH) has also been investigated as a potential intervention for reducing secondary injury following cerebral ischemia and reperfusion. Accumulating evidence suggests that ischemia–reperfusion injury is closely associated with BBB disruption, contributing to vasogenic edema and exacerbation of tissue damage. Experimental and clinical studies have shown that hypothermic conditions can preserve endothelial function, reduce permeability, and stabilize tight junction integrity during ischemic and reperfusion phases [18–20]. However, relatively few in vitro stroke models have specifically focused on the ischemic penumbra, which represents the major therapeutic window in acute ischemic stroke, or incorporated reperfusion-associated recovery processes into BBB-centered experimental systems [15].

In the present study, we developed a 3D BBB chip-based stroke model designed to reproduce penumbra-like, partially reversible ischemic BBB injury. To achieve this, we: (1) established an acute ischemia-like microenvironment using oxygen-glucose deprivation and antimycin A; (2) simulated reperfusion through medium replacement to evaluate BBB recovery under reperfusion-like conditions; and (3) investigated whether therapeutic hypothermia enhances BBB recovery and protective responses in this human in vitro BBB platform.

## Materials and methods

### Cell culture and stroke induction

Primary Human brain microvascular endothelial cells (HBMECs; P10361, Innoprot), primary astrocytes (Cat. #1800, ScienCell), and primary pericytes (Cat. #1200, ScienCell) were cultured in T75 flasks using Endothelial Cell Medium (ECM, #1001, ScienCell), Astrocyte Medium (AM, #1801, ScienCell), and Pericyte Medium (PM, #1201, ScienCell), respectively, each supplemented with the recommended growth factors and antibiotics. Cells were maintained at 37°C in a humidified 5% CO₂ atmosphere and used up to passage 10. Stroke-like injury was induced using antimycin A (A8674, Sigma-Aldrich) in custom glucose-free medium (GFM) at concentrations of 1.25, 2.5, 5, or 10 µM for 1 or 3 hours.

### Preparation of the emulate BBB chip

Microfluidic BBB chips (Chip-S1®, Emulate Inc., Boston, MA, USA) were prepared according to manufacturer protocols [21,22]. Chips were functionalized with ER-1 (#10461) and ER-2 (#10462) solutions, UV-exposed (365 nm, 100 µJ/cm² for 20 minutes), and coated with human extracellular matrix proteins: collagen IV (400 µg/mL; C5533, Sigma), fibronectin (100 µg/mL; F2006, Sigma), and laminin (20 µg/mL; 23017015, Gibco). After coating, HBMECs (4 × 10⁶ cells/mL) were seeded into the lower (vascular) channel of the chip, while astrocytes (5 × 10⁵ cells/mL) and pericytes (2 × 10⁴ cells/mL) were introduced into the upper (parenchymal) channel. The chip was maintained under standard culture conditions (37°C, 5% CO₂), and endothelial monolayer formation was monitored. Gravity-driven perfusion was applied during culture and experimental procedures to provide controlled microfluidic perfusion conditions. Perfusion was driven by a hydrostatic pressure difference between inlet and outlet reservoirs, without the use of external pumps or rocker-based systems. Flow rate and shear stress were not quantitatively measured in this study. The lower (vascular) channel was maintained with endothelial cell medium (ECM), while the upper (parenchymal) channel was supplied with a mixed medium composed of astrocyte and pericyte media at a 1:1 ratio to support the tri-culture system. No additional endothelial maturation protocol was applied. BBB-like properties were induced through the triculture configuration and extracellular matrix coating.

### Preparation of the transwell model

The Transwell model was established using HBMECs, astrocytes, and pericytes, following previously reported protocols [23,24], with modifications tailored to the specific requirements of our experimental conditions. For the triculture model, a mixed suspension of astrocytes (5 × 10⁵ cells/mL) and pericytes (2 × 10⁴ cells/mL) was seeded onto the underside of collagen I–coated Transwell inserts (inverted seeding; 100 µg/mL collagen I, PureCol® EZ Gel #5074). Transwell inserts (polycarbonate membrane, 0.4 µm pore size, 24-well and 6-well formats; SPL Life Sciences, #30024 and #30006, respectively) were used for the co-culture system. After cell attachment, the inserts were returned to an upright position, and HBMECs were seeded on the upper surface of the membrane at 7.15 × 10⁴ cells/cm². The membrane was pre-coated with growth factor–reduced Matrigel (#356230, Corning) prior to HBMEC seeding. This triculture configuration—HBMECs on the upper membrane surface and astrocytes plus pericytes on the underside—was maintained in co-culture conditions to promote the development of BBB properties. Endothelial cell medium (ECM) was added to the upper (insert) compartment, while a mixed medium of astrocyte and pericyte media (1:1) was applied to the lower compartment in contact with astrocytes and pericytes to support the tri-culture system. No additional endothelial maturation protocol was applied, and BBB-like properties were induced under the same co-culture conditions.

### Reperfusion and hypothermia treatment

For reperfusion simulations, cells were returned to normoxic incubation with fresh complete medium after stroke induction. Recovery periods of up to 48 hours were tested to observe cell viability and behavior post-injury. Mild hypothermia treatment was implemented by transferring cultures to a 33 °C incubator immediately after the ischemic insult. Cells in the hypothermia group were maintained at 33 °C throughout the reperfusion period, whereas normothermic controls remained at 37 °C.

### Cell viability assays

Cell viability was assessed using a fluorescent staining cocktail consisting of calcein-AM (1 µM; C1430, Thermo Fisher), propidium iodide (PI, 1 µg/mL; P4564, Sigma-Aldrich), and Hoechst 33342 (10 µg/mL; H3570, Invitrogen). After treatment, live and dead cells were visualized by fluorescence microscopy. Viable cells (calcein-AM–positive, PI-negative) were manually counted in defined microscopic fields, and cell viability was calculated as the percentage of live cells relative to the total number of Hoechst-stained nuclei. All image analyses were performed using consistent evaluation criteria across all experimental conditions to minimize potential observer bias. Automated image analysis was considered; however, overlapping fluorescence signals and variable cell density limited reliable discrimination of individual cells under several experimental conditions.

### Immunocytochemistry

Cells were fixed with 4% paraformaldehyde and permeabilized using 0.1% Triton X-100 in PBS. After blocking with 3% BSA in PBS, samples were incubated with primary antibodies against ZO-1 (1:200; Cat. No. 33−9100, Invitrogen) and claudin-5 (1:200; Cat. No. MA5−32614, Invitrogen). Following PBS washes, secondary antibodies conjugated to Alexa Fluor Plus 488 (A32723) and Alexa Fluor 594 (A11037; Invitrogen) were applied. Nuclei were counterstained with DAPI (D1306, Invitrogen). Samples were mounted using Fluoromount-G (0100−01, SouthernBiotech) and imaged with an Olympus CKX53 fluorescence microscope.

### Western blot analysis

For Western blot analysis, protein lysates were collected exclusively from the HBMECs layer. Although pericytes and astrocytes were co-cultured to support BBB maturation and function, these cells were not harvested for protein extraction. Total proteins were extracted using PRO-PREP lysis buffer (17081, iNtRON Biotechnology) and quantified using a BCA protein assay kit (23227, Thermo Scientific). Equal protein amounts were separated on 12% TGX gels (11–0185, Bio-Rad) and transferred to PVDF membranes (162–0177, Bio-Rad). Membranes were blocked with 5% non-fat dry milk in TBST and incubated with primary antibodies: Anti-HIF-1α (ab1, abcam), anti-RBM3 (MA5–31340, Invitrogen), anti-CIRBP (ab191885, Abcam), and anti-GAPDH (ab9485, Abcam). HRP-conjugated secondary antibodies (anti-mouse: ab6728; anti-rabbit: ab6721; Abcam) were used with WestGlow chemiluminescent substrates (BWF0200, Biomax) for signal detection. Protein bands were imaged using a ChemiDoc XRS+ system (Bio-Rad) and quantified with ImageJ software (NIH).

### TEER measurements

Barrier function was assessed by measuring TEER using an EVOM2 voltohmmeter with STX3 electrodes (World Precision Instruments).

### Permeability assay

Paracellular permeability was assessed using FITC-Dextran tracers with molecular weights of 4 kDa (Cat. #46944–100MG-F, Sigma) and 40 kDa (FD40S-100MG, Sigma), each prepared at a final concentration of 1 mg/mL in culture medium. The tracers were added to the upper chamber of the BBB model, and after incubation, fluorescence in the lower chamber was measured using a SpectraMax M5 plate reader (Molecular Devices) at 485 nm excitation and 538 nm emission wavelengths. The degree of fluorescence detected in the lower compartment was used to quantify paracellular permeability.

### qRT-PCR

Total RNA was extracted from cells using a RNeasy Mini Kit (74101, Qiagen) according to the manufacturer’s instructions. RNA quantity and purity were assessed with a NanoDrop spectrophotometer. cDNA was synthesized from 1 µg of RNA using the ReverTra Ace qPCR RT Master Mix (FSQ-201, TOYOBO). Gene-specific primers (listed in Table 1) were used for PCR amplification with Maxima SYBR Green/ROX qPCR Master Mix (K0222, Thermo Scientific) on a Bio-Rad CFX96 Real-Time PCR System. The PCR program consisted of 50°C for 2 min and 95°C for 10 min, followed by 40 cycles of 95°C for 15 sec and 60°C for 1 min. A melt-curve analysis was performed at the end of the run to confirm primer specificity. Gene expression levels were normalized to GAPDH and calculated using the ΔΔCt method.

**Table 1:** Primer sequences used for quantitative real-time PCR analysis.

Gene name	Gene symbol	GenBank accession no.	Oligonucleotide sequences (5′–3′)
Glyceraldehyde-3-phosphate dehydrogenase	GAPDH	NM_002046	F: GTCTCCTCTGACTTCAACAGCGR: ACCACCCTGTTGCTGTAGCCAA
Zona occludens-1	ZO-1	NM_003257	F: GTCCAGAATCTCGGAAAAGTGCCR: CTTTCAGCGCACCATACCAACC
Occludin	OCLN	NM_002538	F: ATGGCAAAGTGAATGACAAGCGGR: CTGTAACGAGGCTGCCTGAAGT
Vascular Endothelial cadherin	VECAD	NM_001795	F: GAAGCCTCTGATTGGCACAGTGR: TTTTGTGACTCGGAAGAACTGGC

### Final experimental protocol

**Fig 1:**
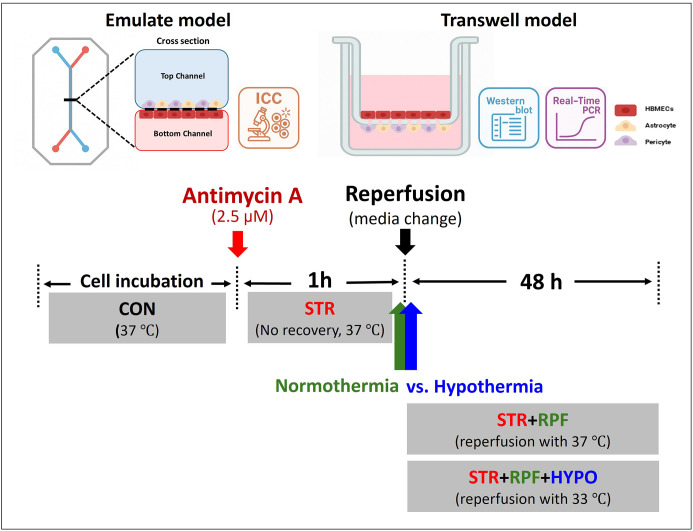
Schematic diagram of the experimental design for modeling acute ischemic injury and penumbra-like BBB dysfunction in 3D BBB platforms. The study was conducted using both the Emulate organ-on-chip and Transwell BBB models. After initial cell incubation under normothermic conditions (37 °C), ischemic stroke was induced by treatment with antimycin A (2.5 μM) for 1 hour (STR). In the STR group, no recovery phase was applied following ischemia. Reperfusion was simulated by media replacement and maintained for 48 hours under either normothermia (37 °C; STR + RPF) or therapeutic hypothermia (33 °C; STR + RPF+HYPO). The CON group remained untreated. BBB integrity and treatment responses were assessed using immunocytochemistry, Western blotting, and qRT-PCR following the 48-hour reperfusion or control incubation period.

Three experimental conditions were established to model ischemic stroke and interventions in vitro. In all conditions, a chemical ischemic insult was induced by treating the blood–brain barrier (BBB) models with antimycin A, a mitochondrial complex III inhibitor, to simulate stroke-like energy deprivation. The conditions differed in post-insult treatment (reperfusion and hypothermia) and temperature (Fig 1):

- Condition A – Stroke Only: Cultures were exposed to antimycin A (ischemic insult) at 37°C for a defined period (1 hour), after which cells were immediately fixed or harvested without any recovery phase. This condition represents an ischemic stroke with no reperfusion, capturing the direct effects of sustained ischemia.
- Condition B – Stroke + Reperfusion: Cultures underwent the same antimycin A ischemic insult (duration and conditions identical to A), but afterward the insult medium was removed and replaced with fresh, oxygenated culture medium to simulate reperfusion. Cells were then allowed to recover under normothermic conditions (37°C) for an additional period (48 hours). Thus, Condition B represents an ischemic event followed by reperfusion at normal body temperature.
- Condition C – Stroke + Reperfusion + Hypothermia: This condition followed the same protocol as Condition B with an initial antimycin A ischemic insult at 37°C, followed by replacement with fresh medium for recovery. However, during the reperfusion/recovery phase, the cultures were maintained under mild hypothermia (33°C) instead of 37°C. The hypothermic treatment was applied immediately at the onset of reperfusion and sustained throughout the recovery period (48 hours), modeling therapeutic hypothermia. Condition C thereby reflects stroke with reperfusion under cooling intervention.

For all groups, the timing of insult and recovery was consistent so that the total experimental duration was the same. Antimycin A was removed by washing during the reperfusion phase in Conditions B and C. By comparing these groups, we assessed the impact of reperfusion alone and reperfusion with hypothermia on BBB integrity, relative to sustained ischemia.

### BBB model configuration

Experiments were conducted using two complementary in vitro blood–brain barrier (BBB) models: organ-on-a-chip system (Emulate BBB Chip) and a conventional Transwell model. Both platforms employed co-cultures of human brain microvascular endothelial cells (HBMECs), supported by astrocytes and pericytes, to mimic the neurovascular unit. The organ-on-a-chip and Transwell models were configured as follows:

Emulate Organ-on-Chip Model: The microfluidic BBB chip (Emulate Inc.) consists of two parallel microchannels (vascular and brain compartments) separated by a porous flexible membrane. A mixed culture of human brain microvascular endothelial cells, astrocytes, and pericytes was established across the membrane to mimic the neurovascular unit. Endothelial cells were seeded on the inner surface of the membrane in the vascular channel, while astrocytes and pericytes were co-cultured in the adjacent brain channel, recreating the blood–brain interface in a microengineered format. The chips were maintained under standard culture conditions (37°C, 5% CO₂). After several days of culture, endothelial monolayer formation and barrier integrity were evaluated. Immunofluorescence staining of tight junction proteins, such as ZO-1, demonstrated continuous junctional patterns outlining the endothelial cells. Fluorescence microscopy revealed well-defined tight junction belts, indicating the successful formation of a confluent and BBB-like barrier within the chip.

Transwell Model: In parallel, Transwell BBB model was established using semi-permeable membrane inserts (polycarbonate membrane, 0.4 µm pore) in multi-well plates. Endothelial cells were cultured on the upper side of the Transwell membrane, with astrocytes and pericytes present in the lower chamber (basal side), allowing diffusive cell–cell signaling. Co-cultures were grown under static conditions at 37°C until a tight barrier formed. To verify BBB formation in Transwells, standard barrier integrity assays were performed. Transendothelial electrical resistance (TEER) was measured across the endothelial monolayer, yielding high TEER values consistent with the formation of a tight barrier. In addition, permeability assays with a FITC-Dextran indicated very low paracellular leakage in the matured monolayers, comparable to literature values for an intact BBB. The combination of high TEER values and low permeability, as shown in Fig S8, confirmed that Transwell co-culture established a functional BBB. This served as a baseline for comparing ischemic injury responses between the Emulate organ-on-chip model and the Transwell insert model.

### Statistical analysis

All quantitative data are presented as the mean ± standard error (SE) of independent samples with n = 3–4 per group. Statistical comparisons were performed using one- or two-way analysis of variance (ANOVA) as appropriate, followed by Tukey’s multiple-comparison post hoc test. Statistical significance was defined as *P < 0.05; **P < 0.01; ***P < 0.001; ****P < 0.0001. All analyses were conducted using GraphPad Prism 7 (GraphPad Software, La Jolla, CA, USA).

## Result

### Cell culture and stroke induction

Preliminary 2D culture experiments were conducted to refine the stroke and treatment protocol for the Transwell/organ-on-chip BBB model. The results below correspond to S1 Fig, which summarizes the findings from these optimization tests. Schematic workflow illustrating the development and validation of in vitro stroke and hypothermia models using 2D and 3D blood–brain barrier (BBB) platforms. In the first phase, a 2D monolayer culture system was optimized to determine the appropriate conditions for ischemic injury and hypothermia-based recovery. Human brain microvascular endothelial cells (HBMECs), astrocytes, and pericytes were individually exposed to antimycin A to simulate stroke, followed by hypothermic treatment to assess cell-type specific responses. Based on these results, two types of 3D BBB models—a microfluidic organ-on-chip system (Emulate Brain-Chip) and a conventional Transwell co-culture—were constructed using the same cell types to replicate the neurovascular unit. Functional validation of the models was performed via immunocytochemistry (ICC) for tight junction proteins, transendothelial electrical resistance (TEER) measurement, and FITC-Dextran permeability assays.

### Stroke induction in 2D monocultures

Treatment with antimycin A for 1 hour caused a significant decrease in cell viability across all three cell types. HBMECs, astrocytes, and pericytes each showed markedly reduced viable cell fractions compared to untreated controls, confirming effective induction of ischemic injury. This 1-hour chemical ischemia condition (2.5 μM antimycin A) was therefore selected as the optimal stroke induction, as shorter exposures caused minimal cell death while longer exposures did not further reduce viability (S2, S3 Figs).

### Endothelial tight junction disruption

We next confirmed that the stroke-mimicking insult compromises endothelial barrier properties. Immunofluorescence staining of tight junction proteins in HBMECs revealed clear differences between control and stroke conditions (S4A Fig). In normal (untreated) endothelial cultures, ZO-1 and claudin-5 were continuously localized at cell–cell borders, indicating intact cell junctions. In contrast, endothelial cells fixed immediately after the 1-hour antimycin A treatment showed discontinuous and patchy ZO-1/claudin-5 staining (S4B Fig). This loss of continuous junctional signal confirms that our *in vitro* stroke conditions disrupt endothelial tight junction integrity, mimicking blood–brain barrier (BBB) breakdown.

### Cell recovery during reperfusion

The ability of cells to recover after ischemic injury was assessed by reintroducing normal culture conditions for 48 hours post-insult. Notably, HBMEC cultures that had undergone the 1-hour stroke induction were able to re-form a confluent monolayer after 48 hours of reperfusion. Phase-contrast microscopy at 48 hours showed that previously injured endothelial cells spread and regained cell–cell contacts, although the monolayer remained slightly less uniform compared to uninjured controls (S5 Fig). This indicates partial recovery of the endothelial layer with extended reperfusion, supporting the inclusion of a prolonged recovery phase in the protocol.

### Hypothermia treatment efficacy

Mild hypothermia during reperfusion had a visibly protective effect on cells following stroke injury. When cultures were maintained at 33 °C for the 48-hour reperfusion period, cell morphology and coverage were improved relative to the normothermic reperfusion condition. Endothelial cells in the hypothermia group appeared more polygonal and tightly packed, similar to uninjured controls, whereas cells at 37 °C showed areas of cell thinning. Moreover, qualitative viability appeared higher with 33 °C treatment, suggesting reduced delayed cell death. These observations validated 33 °C as an effective therapeutic hypothermia condition, which was incorporated into the final protocol (S6 Fig).

### Cell viability assays in the Emulate organ-on-chip model

The cytotoxic response of the Emulate organ-on-chip BBB model to antimycin A–induced ischemic injury was assessed using a Calcein-AM/PI live/dead assay. Endothelial cell viability decreased in a dose-dependent manner with increasing concentrations of antimycin A. Treatment with 1.25 μM resulted in 96.1% viability, while 2.5 μM, 5 μM, and 10 μM led to reduced viability levels of 68.6%, 43.2%, and 24.9%, respectively. These findings indicate a sharp decline in viability at concentrations above 2.5 μM, suggesting that 2.5 μM represents a threshold dose for partially reversible ischemic damage in the BBB chip model (S7 Fig).

### Emulate Organ-on-chip model

**Fig 2:**
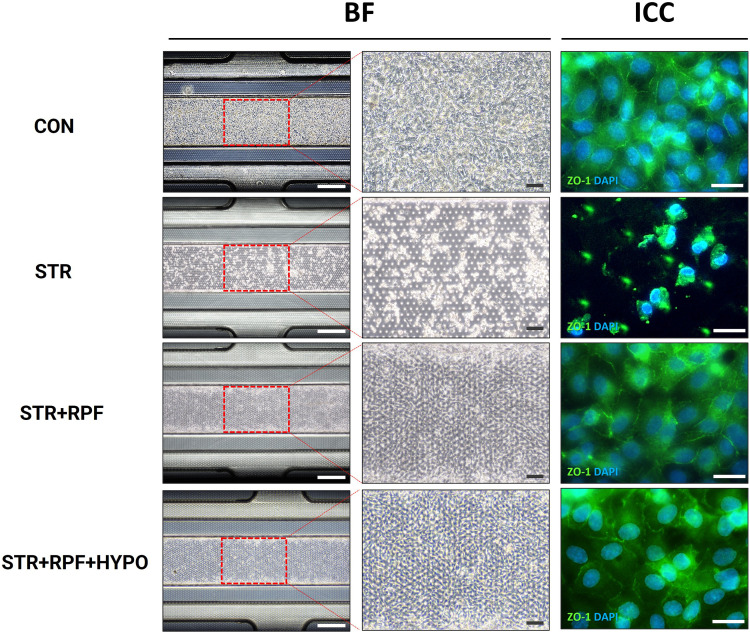
Bright-field and immunocytochemical analysis of endothelial cells in the Emulate organ-on-chip model under ischemic and penumbra-like recovery conditions. Bright-field and immunocytochemical analysis of endothelial cells in the Emulate organ-on-chip model under ischemic and penumbra-like recovery conditions. Human brain microvascular endothelial cells were evaluated under four conditions: CON, STR, STR + RPF, and STR + RPF+HYPO. Bright-field (BF) images show disruption of the endothelial monolayer in the STR group, which was partially restored in the STR + RPF group and further improved in the STR + RPF+HYPO group. Immunocytochemistry (ICC) images demonstrate tight junction protein ZO-1 (green) and nuclear counterstaining with DAPI (blue). Restoration of continuous ZO-1 expression was observed following reperfusion and hypothermia treatment. Scale bars = 500 μm (left, low-magnification BF), 100 μm (middle, high-magnification BF), and 25 μm (right, ICC). Images are representative of three independent experiments (n = 3 independent chips per condition).

Tight Junction Integrity by ZO-1 Immunofluorescence: In the organ-on-chip BBB model, ZO-1 immunofluorescence microscopy revealed distinct differences in tight junction integrity across the three experimental conditions (A, B, C). Under the stroke-only condition A, the endothelial tight junctions were markedly disrupted. ZO-1 staining in Condition A appeared discontinuous and fragmented, with gaps between cells, indicating significant compromise of the junctional complexes after the ischemic insult. In contrast, Condition B (Stroke + Reperfusion) showed partial restoration of ZO-1 localization at cell–cell borders. Following 48 hours of reperfusion, the endothelial monolayer in the chip exhibited more continuous ZO-1 labeling than in Condition A, although some discontinuities remained. These findings suggest that reperfusion with restoration of oxygen and nutrients at 37 °C partially promoted recovery of tight junction integrity following ischemic injury. Notably, the hypothermia-treated group (Condition C) retained the most intact ZO-1 structure. When reperfusion was conducted under mild hypothermia, ZO-1 immunostaining was largely continuous, with belt-like junctional patterns like uninjured control chips. Fewer gaps or disruptions were observed in Condition C, indicating that cooling during reperfusion preserved tight junction integrity far better than in Condition B. Representative fluorescent images for each group are shown in Fig 2, illustrating these qualitative differences: the stroke-only chip has blurred or faint junctional lines, the normothermic reperfusion chip shows moderate ZO-1 reassembly, and the hypothermic reperfusion chip displays bright, continuous ZO-1 outlining each cell. In summary, the organ-on-chip results demonstrate that while ischemia alone severely compromises BBB tight junctions, introducing a reperfusion phase helps regain some barrier function, and applying therapeutic hypothermia during reperfusion yields an even more pronounced protective effect on tight junction preservation.

### Transwell model

**Fig 3:**
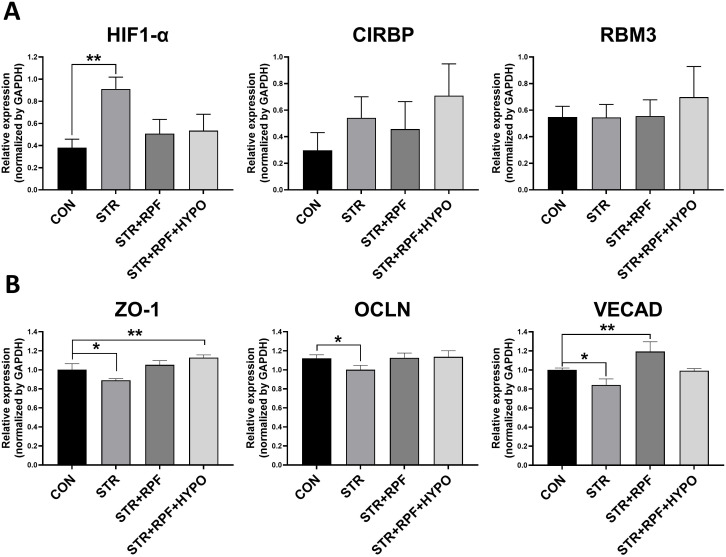
Assessment of tight junction and hypothermia-related markers in the Emulate BBB chip under different experimental conditions. (A) Western blot analysis of HIF-1α, CIRBP, and RBM3 expression. Representative blots for HIF-1α, CIRBP, RBM3, and GAPDH (loading control) are shown under each experimental condition (NOR, STR, STR + RPF, STR+HYPO, and STR + RPF+HYPO). Densitometric quantification of protein levels normalized to GAPDH is presented as mean ± SD (n = 4 per group). HIF-1α expression was significantly elevated in the STR group compared with NOR and decreased under reperfusion and hypothermia conditions. CIRBP and RBM3 expression showed increasing trends under hypothermic conditions, with RBM3 showing the highest expression in the STR + RPF+HYPO group. (B) qRT-PCR analysis of BBB-related gene expression in the Transwell model following ischemic stroke and treatment. ZO-1 mRNA expression was significantly increased in the STR + RPF+HYPO group, and VECAD (VE-cadherin) mRNA expression was significantly increased in the STR + RPF group compared with NOR. Data are presented as mean ± SD (n = 4 per group). *P < 0.05, **P < 0.01 (one-way ANOVA with Tukey’s post hoc test). These molecular changes are consistent with recovery of a penumbra-like, partially reversible BBB injury state..

Hypoxia- and Hypothermia-Related Protein Expression – Western Blot: In the Transwell BBB model, Western blot analysis was performed to evaluate the expression of HIF-1α, CIRBP, and RBM3 under different experimental conditions (Fig 3A). HIF-1α expression was markedly increased under the STR condition, indicating successful induction of hypoxia-/ischemia-like metabolic stress by antimycin A treatment. In contrast, HIF-1α expression showed decreasing trends under reperfusion conditions (STR + RPF and STR + RPF+HYPO), suggesting partial alleviation of ischemic stress following reperfusion.CIRBP and RBM3, representative cold shock proteins associated with hypothermic responses, showed limited changes under the STR condition alone but exhibited increased expression trends under hypothermic reperfusion conditions (STR + RPF+HYPO). In particular, RBM3 expression was highest under hypothermic conditions, supporting its role as a representative marker of cold stress response.These findings demonstrate that the present model successfully reproduced both ischemia-associated stress responses and hypothermia-related molecular responses at the protein level. Accordingly, Fig 3A provides complementary protein-level evidence supporting induction of ischemic stress (HIF-1α) and hypothermic responses (CIRBP and RBM3) in the current BBB stroke model.

Tight Junction mRNA Levels – qRT-PCR: Gene expression analyses by quantitative RT-PCR mirrored the protein findings (Fig 3B). mRNA transcripts encoding tight junction and adherens junction proteins were down-regulated by ischemic injury and differentially restored by reperfusion and hypothermia. In Condition A (stroke only), transcript levels for ZO-1 (TJP1 gene), occludin (OCLN), and VE-Cadherin (VECAD) were all significantly lower than in control inserts, indicating reduced gene expression concomitant with junctional damage. Condition B (reperfusion) samples showed higher mRNA levels for these genes compared to Condition A, suggesting that the restoration of oxygen and nutrients during reperfusion promoted the upregulation of tight junction components as cells attempted to repair the barrier. However, the mRNA levels in normothermic reperfusion were still moderately depressed relative to controls. Condition C (hypothermic reperfusion) demonstrated the strongest recovery in gene expression. ZO-1, OCLN, and VECAD mRNA in the hypothermia group were significantly elevated in comparison to the normothermic group, in many cases not statistically different from control levels. This indicates that hypothermia not only preserved junctional proteins but also supported the maintenance or re-initiation of gene expression programs for barrier constituents after ischemic stress.

Collectively, the Transwell model results align with the organ-on-chip findings. Both protein and mRNA data show that an ischemic insult alone severely impairs the BBB, reperfusion provides partial restoration of barrier elements, and the addition of therapeutic hypothermia during reperfusion yields a markedly improved preservation of BBB integrity. Overall, the concordant results from the two BBB models strengthen the conclusion that hypothermic treatment during post-stroke reperfusion significantly attenuates BBB disruption, helping to maintain tight junction structure and function.

## Discussion

This study is noteworthy in that we used a human cell-based 3D BBB model to examine the pathophysiology and treatment responses of acute ischemic stroke. We confirmed the model’s potential as a preclinical platform capable of simulating an ischemic penumbra and incorporating treatment interventions (reperfusion and therapeutic hypothermia).

### Optimized conditions enhance model viability and reproducibility

For effective replication of an ischemic penumbra in an acute ischemic stroke model, the injury to cells must not be excessive, so that a reversible condition can be induced. In this study, we optimized the injury conditions for each cell type to enhance the viability and reproducibility of our model. Using a 2D culture-based model, we titrated the antimycin A concentration and exposure time to identify the optimal acute ischemic stroke conditions (see S1–S3 Figs). We employed a calcein-AM/propidium iodide (PI) live-dead assay to guide optimization of the ischemic penumbra conditions. This assay measures cell survival and death based on membrane integrity and esterase activity, providing an intuitive, image-based readout of cell viability (see S2 Fig) [25].

Additionally, to simulate reperfusion, we replaced the culture medium with fresh medium after antimycin A treatment. The hypothermia treatment was applied by incubating cells at 33°C for 48 hours. Through this cell type-specific optimization approach, we achieved stroke injury conditions that improved model viability and reproducibility while more realistically reflecting a clinically observed ischemic penumbra.

### Clinically relevant model encompassing ischemic penumbra, reperfusion, and hypothermia

For an acute ischemic stroke chip to be useful in preclinical research on pathophysiology and new therapies, it must include salvageable tissue (ischemic penumbra) like what is seen in animal models [26]. Moreover, the method of inducing ischemic injury should have a high success rate and reflect the physiological conditions of human stroke patients [17,27]. Existing approaches for creating stroke conditions in vitro fall into two categories: using hypoxic chambers or GasPak systems to lower oxygen levels in the culture environment [15,24,27] and using pharmacological agents like antimycin A to inhibit mitochondrial complex III, thereby reducing ATP production and oxygen consumption to induce a hypoxic state [28,29]. The hypoxic chamber approach generally requires over 24 hours of oxygen deprivation to induce ischemia [15], which may not mirror clinical reality given that salvageable tissue is largely absent beyond ~6 hours after stroke onset in patients. In contrast, the drug-based approach can induce ischemic injury in a shorter time and allows precise control over the severity and duration of the insult.

In a previous BBB chip study, 10 µM antimycin A for 16 hours was used to induce ischemic stroke conditions [9]. However, in our model this condition drastically reduced cell viability, effectively creating a permanent ischemic injury. We therefore tested antimycin A at 1.25–10 µM on our triple co-culture (HBMECs, astrocytes, pericytes) and identified 2.5 µM for 1 hour as the optimal condition, which yielded 82–98% cell viability (see S7 Fig). This treatment established a partially recoverable ischemic penumbra state. To our knowledge, this is the first study to optimize an antimycin A–based BBB chip model, suggesting a new approach for precisely simulating an ischemic penumbra. An exposure time of 1 hour was selected to better replicate the transient and partially reversible characteristics of the ischemic penumbra, thereby allowing for potential barrier recovery. S5 Fig shows representative phase-contrast images of HBMECs in 2D culture following antimycin A–induced stroke and reperfusion, confirming partial morphological recovery by 48 hours.

Using these optimized conditions, we built a model with a high induction success rate that closely mimics clinical stroke conditions, effectively reproducing acute ischemic injury. Notably, the same conditions were applied in the Emulate model, where we observed 69–90% cell viability along with decreased ZO-1 expression and enlarged intercellular gaps, indicating clear BBB disruption under our acute stroke and reperfusion model (see S4 and S7 Fig). In this study, reperfusion was experimentally modeled by replacing the culture medium after ischemic insult; however, this approach does not fully recapitulate clinical reperfusion. Media replacement enables controlled re-supply of oxygen and nutrients following ischemia, allowing reproducible evaluation of barrier recovery under simplified conditions. Nevertheless, such experimental conditions do not encompass the full complexity of reperfusion observed in vivo. Therefore, reperfusion in this system should be interpreted as a simplified experimental approximation rather than a direct representation of the clinical condition [30–32].

The ischemic model used in the present study does not directly recapitulate hemodynamic features such as blood flow reduction, shear stress alterations, or vascular occlusion. Instead, the model was designed to induce metabolic stress using antimycin A and glucose-free medium to reproduce cellular energy depletion and functional impairment associated with ischemic conditions. This approach enables precise control of ischemic severity and duration, which is advantageous for reproducibly inducing a partially recoverable ischemic penumbra-like state. Therefore, the present model should be interpreted primarily as a metabolic ischemia model for investigating BBB injury and recovery processes rather than a flow-based mechanical ischemia model. Nevertheless, the absence of hemodynamic components remains a limitation of the current system, and incorporation of flow-based ischemic conditions should be considered in future studies.

### Enabling analysis of combined reperfusion and hypothermia effects

In acute ischemic stroke treatment, rapid reperfusion is critical, and a precisely controllable and reproducible experimental system is required to evaluate its effects [26,33]. In the present study, reperfusion was simulated by replacing the culture medium immediately after induction of ischemic injury. Therapeutic hypothermia conditions were also optimized prior to the main experiments. Preliminary experiments performed under multiple temperature conditions (31 °C, 33 °C, and 35 °C) demonstrated that 33 °C induced the most prominent expression of the cold shock proteins RBM3 and CIRBP (S6 Fig). Based on these findings, 33 °C was selected as the representative therapeutic hypothermia condition for the subsequent experiments. This observation is consistent with previous reports supporting 33 °C as an effective temperature for BBB protection and neuroprotective responses [34–37].

Under these optimized conditions, the combined reperfusion plus hypothermia group (STR + RPF+HYPO) showed the most pronounced recovery of tight junction-related markers, including ZO-1 and VE-cadherin, suggesting a synergistic protective effect of the combined treatment on BBB recovery. These findings indicate that the present platform can be used to evaluate combined reperfusion- and hypothermia-associated BBB recovery responses.

Moreover, HIF-1α, a representative marker of hypoxic stress, was significantly elevated in the stroke-only (STR) group compared with the reperfusion group (STR + RPF), supporting that the model successfully reproduced ischemia-like metabolic stress conditions. Previous studies have shown that HIF-1-related pathways may also be activated under pseudohypoxic conditions associated with mitochondrial dysfunction, even in the absence of true hypoxia [38,39]. Under metabolic inhibition conditions such as mitochondrial complex III blockade by antimycin A, cells can experience a functionally hypoxia-like state that induces HIF-1α-related responses [40–42].

In addition, previous studies have described the ischemic penumbra as a metabolically stressed but partially recoverable state characterized by mitochondrial dysfunction, ATP depletion, and increased ROS accumulation [43–45]. In this context, the partial recovery patterns observed in the present study may be interpreted as physiologically consistent with previously described features of ischemic penumbra-like injury, although direct metabolic functional validation was not performed in the current work.

### Improved structural and functional completeness by combining chip and Transwell models

The Emulate model used in this study is a commercially available platform engineered to closely mimic the human microenvironment and support human-relevant cellular responses [46–48]. Our results demonstrated that both the Emulate BBB chip and the Transwell model successfully reproduced key structural and functional characteristics of the BBB using a triple co-culture system consisting of HBMECs, astrocytes, and pericytes. Increased expression of tight junction-associated proteins, elevated transendothelial electrical resistance (TEER), and reduced FITC-Dextran permeability collectively supported successful BBB formation in these models (see S8 Fig). Notably, the Emulate BBB chip enabled endpoint-based structural evaluation of cellular and tight junction responses under acute ischemia- and reperfusion-like conditions, providing complementary morphological information alongside the functional and molecular analyses performed using the Transwell model. Although some differences were observed between the two platforms, these findings likely reflect differences between structural and molecular assessments rather than directly contradictory results [49]. The Emulate BBB chip was primarily used for structural evaluation of cellular and tight junction responses, whereas the Transwell model was used to complement the study with molecular and functional analyses. Therefore, the findings obtained from the two systems should be interpreted as complementary assessments reflecting different aspects of BBB injury and recovery.

In the Emulate BBB chip, ZO-1 was primarily used as the representative tight junction marker for structural evaluation because of experimental limitations associated with multiplex imaging and sample handling. Barrier-related molecular changes were complementarily assessed using qRT-PCR analyses of ZO-1, OCLN, and VECAD expression. Previous studies have suggested that BBB integrity is regulated through the interaction of multiple tight junction-associated proteins, including claudin-5, and that BBB characterization is commonly based on multiple complementary markers rather than a single marker alone [50,51].

The Emulate BBB chip enabled endpoint-based structural evaluation of BBB injury and recovery under ischemia- and reperfusion-like conditions.

However, the Emulate model also has several limitations, including high cost and limited cell capacity, which made it difficult to obtain sufficient sample volumes for extensive molecular analyses such as Western blotting and qRT-PCR [48]. In addition, stable TEER measurement is technically challenging in the Emulate BBB chip because of its closed microfluidic structure and lack of integrated electrodes. Therefore, BBB integrity in the Emulate system was evaluated using FITC-Dextran permeability assays and tight junction immunofluorescence analyses. Under baseline BBB maturation conditions, permeability to both 4 kDa and 40 kDa FITC-Dextran remained low, with 4 kDa permeability measuring approximately 8–12% of the no-cell control level, supporting successful BBB formation. These findings showed trends consistent with the low permeability and elevated TEER values observed in the Transwell model. However, permeability changes under ischemic and reperfusion conditions were not included in the present study and should be addressed in future investigations.

To overcome the limitations of the Emulate system, parallel experiments were performed using the Transwell model, which enabled quantitative molecular and functional analyses, including TEER measurement, protein expression, and gene expression analyses under different stroke conditions (see S1 Table) [24]. The complementary use of these two systems enhanced the overall interpretability and versatility of the present BBB stroke model.

This study has several limitations that should be considered when interpreting the findings. First, the protective effect of therapeutic hypothermia (TH) alone was not statistically significant. Although TH has been shown in previous studies to exert neuroprotective effects [35–37], in our model the benefit may have been diminished by the experimental setup. Specifically, the implementation of reperfusion through medium exchange could have washed out cytokines and other cellular injury signals generated during ischemia. These factors may be important mediators of TH’s therapeutic action, and their removal may have masked any measurable benefit from hypothermia in the absence of reperfusion. Second, our 3D BBB model did not include microglia, the resident immune cells of the central nervous system. While our model incorporated key BBB-associated cell types (HBMECs, astrocytes, and pericytes), the lack of microglia limited our ability to investigate neuroinflammatory responses. Microglia play a central role in mediating inflammation, injury repair, and communication with other neural and vascular cell types during and after ischemic stroke. Without this component, the model cannot fully replicate the inflammatory environment of stroke or be used to evaluate immunomodulatory therapies [52]. Third, the study focused solely on the acute phase of ischemic stroke, with observations limited to 48 hours post-insult. While this time window is appropriate for modeling the ischemic penumbra and assessing immediate treatment effects, it does not capture longer-term outcomes such as delayed injury, sustained BBB recovery, or secondary neurovascular damage. Future studies will need to extend the observational period and incorporate additional components—such as microglia and dynamic perfusion—to better simulate chronic stroke conditions and evaluate the durability of therapeutic interventions. The Emulate Organ-on-Chip system lacks built-in electrodes, and the closed microfluidic design makes it technically challenging to insert external electrodes for reliable TEER measurements. Additionally, bidirectional flow in both channels can interfere with electrical stability. Therefore, barrier integrity in the Emulate model was evaluated indirectly using immunocytochemistry instead [53]. Fluorescence imaging was limited in the Transwell BBB model due to the low optical clarity and light-scattering properties of the insert membranes. Both polyethylene terephthalate (PET) and polycarbonate (PC) membranes are suboptimal for high-resolution fluorescence microscopy; PET membranes cause significant light scattering, while PC membranes are translucent and provide poor visibility of cell outlines. These characteristics hinder clear visualization of cells on both the apical and basolateral surfaces, making accurate imaging of cellular morphology and junctional proteins challenging [54]. Despite these limitations, the model provides a valuable platform for early-phase stroke research.

## Conclusions

Using our human cell–based 3D BBB chip model, we successfully simulated acute ischemic stroke incorporating a reversible penumbra. This platform enables the evaluation of reperfusion and therapeutic hypothermia under physiologically relevant conditions, offering a human-relevant and translational platform to study BBB integrity and stroke recovery. Our model may provide a useful platform for investigating stroke pathophysiology and for screening therapeutic strategies targeting the salvageable penumbra, including barrier-protective or neurovascular-targeted agents and combination therapies.

## Supporting information

Supplementary Materials 1 Supporting Information captions for all supporting figures, tables, files, and datasets. (DOCX)

S1 Fig Optimization of 2D Culture-Based Model: Acute ischemic conditions were induced in a 2D culture by antimycin A. (JPG)

S2 Fig Cell viability assay following antimycin A induced ischemic injury in 2D culture. (JPG)

S3 Fig Quantitative analysis of cell viability assay after ischemic injury. (JPG)

S4 Fig Morphological alterations and disruption of tight junctions in 2D cultured HBMECs under antimycin A induced ischemic conditions. (JPG)

S5 Fig Morphological changes in HBMECs after antimycin A–induced ischemia and reperfusion in 2D culture. (JPG)

S6 Fig Western blot analysis of RBM3 and CIRBP expression in 2D-cultured HBMECs, astrocytes, and pericytes under hypothermic conditions. (JPG)

S7 Fig Cell viability assessment in the Emulate BBB chip following acute ischemic stroke induction. (JPG)

S8 Fig Barrier characterization of the Transwell model. (JPG)

S1 Table Final experimental conditions for the in vitro ischemia–reperfusion injury model (total duration 48 h). (PDF)

S1 Dataset Raw viability data corresponding to S3A Fig. (XLSX)

S2 Dataset Raw viability data corresponding to S3B Fig. (XLSX)

S3 Dataset Raw viability data corresponding to S3C Fig. (XLSX)

S4 Dataset Raw western blot quantification data corresponding to S6A Fig. (XLSX)

S5 Dataset Raw western blot quantification data corresponding to S6B Fig. (XLSX)

S6 Dataset Raw viability data corresponding to S7B Fig. (XLSX)

S7 Dataset Raw FITC-dextran permeability data corresponding to S8C Fig. (XLSX)

S8 Dataset Raw TEER measurement data corresponding to S8B Fig. (XLSX)

S9 Dataset Raw data for CIRBP western blot quantification shown in Fig 3A. (XLSX)

S10 Dataset Raw data for HIF-1α western blot quantification shown in Fig 3A. (XLSX)

S11 Dataset Raw data for RBM3 western blot quantification shown in Fig 3A. (XLSX)

S12 Dataset Raw data for real-time PCR analysis shown in Fig 3C. (XLSX)

S13 File Uncropped western blot image for RBM3 used in S6C Fig. (JPG)

S14 File Uncropped western blot image for CIRBP used in S6C Fig. (JPG)

S1 File Uncropped western blot image for GAPDH used in S6C Fig. (JPG)

S2 File Uncropped western blot image for RBM3 used in Fig 3A. (TIFF)

S3 File Uncropped western blot image for GAPDH used in Fig 3A. (TIFF)

S4 File Uncropped western blot image for CIRBP used in Fig 3A. (TIFF)

S5 File Uncropped western blot image for HIF-1α used in Fig 3A. (TIFF)

## References

[1] Katan M, Luft A. Global burden of stroke. Semin Neurol. 2018;38(2):208–11. doi: 10.1055/s-0038-1649503 29791947

[2] GBD 2015 Neurological Disorders Collaborator Group. Global, regional, and national burden of neurological disorders during 1990-2015: a systematic analysis for the Global Burden of Disease Study 2015. Lancet Neurol. 2017;16(11):877–97. doi: 10.1016/S1474-4422(17)30299-5 28931491 PMC5641502

[3] Macrae IM. Preclinical stroke research--advantages and disadvantages of the most common rodent models of focal ischaemia. Br J Pharmacol. 2011;164(4):1062–78. doi: 10.1111/j.1476-5381.2011.01398.x 21457227 PMC3229752

[4] Woodruff TM, Thundyil J, Tang S-C, Sobey CG, Taylor SM, Arumugam TV. Pathophysiology, treatment, and animal and cellular models of human ischemic stroke. Mol Neurodegener. 2011;6(1):11. doi: 10.1186/1750-1326-6-11 21266064 PMC3037909

[5] Narayan SK, Grace Cherian S, Babu Phaniti P, Babu Chidambaram S, Rachel Vasanthi AH, Arumugam M. Preclinical animal studies in ischemic stroke: Challenges and some solutions. Animal Model Exp Med. 2021;4(2):104–15. doi: 10.1002/ame2.12166 34179718 PMC8212819

[6] Fluri F, Schuhmann MK, Kleinschnitz C. Animal models of ischemic stroke and their application in clinical research. Drug Des Devel Ther. 2015;9:3445–54. doi: 10.2147/DDDT.S56071 26170628 PMC4494187

[7] Heiss WD, Graf R. The ischemic penumbra. Curr Opin Neurol. 1994;7(1):11–9. doi: 10.1097/00019052-199402000-00004 8173671

[8] Taha A, Bobi J, Dammers R, Dijkhuizen RM, Dreyer AY, van Es A, et al. Comparison of large animal models for acute ischemic stroke: which model to use?. Stroke. 2022;53(4):1411–22. doi: 10.1161/STROKEAHA.121.036050 35164533 PMC10962757

[9] Wevers NR, Nair AL, Fowke TM, Pontier M, Kasi DG, Spijkers XM, et al. Modeling ischemic stroke in a triculture neurovascular unit on-a-chip. Fluids Barriers CNS. 2021;18(1):59. doi: 10.1186/s12987-021-00294-9 34906183 PMC8670153

[10] Doke SK, Dhawale SC. Alternatives to animal testing: A review. Saudi Pharm J. 2015;23(3):223–9. doi: 10.1016/j.jsps.2013.11.002 26106269 PMC4475840

[11] Kim S, Kim M, Grant GA, Lee W. The neurovascular unit-on-a-chip: modeling ischemic stroke to stem cell therapy. Neural Regen Res. 2024;19(7):1431–2. doi: 10.4103/1673-5374.385296 38051882 PMC10883497

[12] O’Grady BJ, McCall AS, Cullison S, Chavarria D, Schrag MS, Lippmann ES. Human Brain Vasculature-on-a-Chip Model Constructed With Microvessels Isolated From Cryopreserved Postmortem Human Brain Tissue. Adv Healthc Mater. 2026;15(13):e04167. doi: 10.1002/adhm.202504167 41527268 PMC13058772

[13] Liu Z, Tang Y, Zhang Z, Liu Q, Wang M, Li W, et al. Engineering Neurovascular Unit and Blood-Brain Barrier for Ischemic Stroke Modeling. Adv Healthc Mater. 2023;12(19):e2202638. doi: 10.1002/adhm.202202638 37075477

[14] Park T-E, Mustafaoglu N, Herland A, Hasselkus R, Mannix R, FitzGerald EA, et al. Hypoxia-enhanced Blood-Brain Barrier Chip recapitulates human barrier function and shuttling of drugs and antibodies. Nat Commun. 2019;10(1):2621. doi: 10.1038/s41467-019-10588-0 31197168 PMC6565686

[15] Lyu Z, Park J, Kim K-M, Jin H-J, Wu H, Rajadas J, et al. A neurovascular-unit-on-a-chip for the evaluation of the restorative potential of stem cell therapies for ischaemic stroke. Nat Biomed Eng. 2021;5(8):847–63. doi: 10.1038/s41551-021-00744-7 34385693 PMC8524779

[16] Ahn SI, Sei YJ, Park H-J, Kim J, Ryu Y, Choi JJ, et al. Microengineered human blood-brain barrier platform for understanding nanoparticle transport mechanisms. Nat Commun. 2020;11(1):175. doi: 10.1038/s41467-019-13896-7 31924752 PMC6954233

[17] Pang B, Wu L, Peng Y. In vitro modelling of the neurovascular unit for ischemic stroke research: Emphasis on human cell applications and 3D model design. Exp Neurol. 2024;381:114942. doi: 10.1016/j.expneurol.2024.114942 39222766

[18] Yenari MA, Han HS. Neuroprotective mechanisms of hypothermia in brain ischaemia. Nat Rev Neurosci. 2012;13(4):267–78. doi: 10.1038/nrn3174 22353781

[19] Kaur C, Ling EA. Blood brain barrier in hypoxic-ischemic conditions. Curr Neurovasc Res. 2008;5(1):71–81. doi: 10.2174/156720208783565645 18289024

[20] Baumann E, Preston E, Slinn J, Stanimirovic D. Post-ischemic hypothermia attenuates loss of the vascular basement membrane proteins, agrin and SPARC, and the blood-brain barrier disruption after global cerebral ischemia. Brain Res. 2009;1269:185–97. doi: 10.1016/j.brainres.2009.02.062 19285050

[21] Emulate I. Basic Research Kit Protocol. EP223 Rev. Boston, MA: Emulate; 2022.

[22] Huh D, Kim HJ, Fraser JP, Shea DE, Khan M, Bahinski A, et al. Microfabrication of human organs-on-chips. Nat Protoc. 2013;8(11):2135–57. doi: 10.1038/nprot.2013.137 24113786

[23] Fattakhov N, Torices S, Becker S, Teglas T, Naranjo O, Toborek M. A Triple Primary Cell Culture Model of the Human Blood-Brain Barrier for Studying Ischemic Stroke In Vitro. J Vis Exp. 2022;188:e64469. doi: 10.3791/64469 36282703

[24] Stone NL, England TJ, O’Sullivan SE. A novel transwell blood brain barrier model using primary human cells. Front Cell Neurosci. 2019;13:230. doi: 10.3389/fncel.2019.00230 31244605 PMC6563620

[25] Fan L, Zhang C-J, Zhu L, Chen J, Zhang Z, Liu P, et al. FasL-PDPK1 Pathway Promotes the Cytotoxicity of CD8+ T Cells During Ischemic Stroke. Transl Stroke Res. 2020;11(4):747–61. doi: 10.1007/s12975-019-00749-0 32036560

[26] Thalerová S, Vítečková Wünschová A, Kittová P, Vašátková L, Pešková M, Volný O, et al. A collateral circulation in ischemic stroke accelerates recanalization due to lower clot compaction. PLoS One. 2024;19(11):e0314079. doi: 10.1371/journal.pone.0314079 39561145 PMC11575800

[27] Van Breedam E, Ponsaerts P. Promising Strategies for the Development of Advanced In Vitro Models with High Predictive Power in Ischaemic Stroke Research. Int J Mol Sci. 2022;23(13):7140. doi: 10.3390/ijms23137140 35806146 PMC9266337

[28] Kurian GA, Pemaih B. Standardization of in vitro cell-based model for renal ischemia and reperfusion injury. Indian J Pharm Sci. 2014;76(4):348–53. 25284933 PMC4171872

[29] Kuriakose D, Xiao Z. Pathophysiology and Treatment of Stroke: Present Status and Future Perspectives. Int J Mol Sci. 2020;21(20):7609. doi: 10.3390/ijms21207609 33076218 PMC7589849

[30] Dirnagl U, Iadecola C, Moskowitz MA. Pathobiology of ischaemic stroke: an integrated view. Trends Neurosci. 1999;22(9):391–7. doi: 10.1016/s0166-2236(99)01401-0 10441299

[31] Iadecola C, Anrather J. The immunology of stroke: from mechanisms to translation. Nat Med. 2011;17(7):796–808. doi: 10.1038/nm.2399 21738161 PMC3137275

[32] Hossmann KA. Viability thresholds and the penumbra of focal ischemia. Ann Neurol. 1994;36(4):557–65. doi: 10.1002/ana.410360404 7944288

[33] Ospel J, Rex N, Kandregula S, Goyal M. The vessel has been recanalized: now what?. Neurotherapeutics. 2023;20(3):679–92. doi: 10.1007/s13311-023-01367-3 37014594 PMC10275828

[34] Polderman KH. Mechanisms of action, physiological effects, and complications of hypothermia. Crit Care Med. 2009;37(7 Suppl):S186-202. doi: 10.1097/CCM.0b013e3181aa5241 19535947

[35] Ávila-Gómez P, Vieites-Prado A, Dopico-López A, Bashir S, Fernández-Susavila H, Gubern C, et al. Cold stress protein RBM3 responds to hypothermia and is associated with good stroke outcome. Brain Commun. 2020;2(2):fcaa078. doi: 10.1093/braincomms/fcaa078 33585816 PMC7869850

[36] Jackson TC, Manole MD, Kotermanski SE, Jackson EK, Clark RSB, Kochanek PM. Cold stress protein RBM3 responds to temperature change in an ultra-sensitive manner in young neurons. Neuroscience. 2015;305:268–78. doi: 10.1016/j.neuroscience.2015.08.012 26265550 PMC4570027

[37] Tong G, Endersfelder S, Rosenthal L-M, Wollersheim S, Sauer IM, Bührer C, et al. Effects of moderate and deep hypothermia on RNA-binding proteins RBM3 and CIRP expressions in murine hippocampal brain slices. Brain Res. 2013;1504:74–84. doi: 10.1016/j.brainres.2013.01.041 23415676

[38] Hayashi Y, Yokota A, Harada H, Huang G. Hypoxia/pseudohypoxia-mediated activation of hypoxia-inducible factor-1α in cancer. Cancer Sci. 2019;110(5):1510–7. doi: 10.1111/cas.13990 30844107 PMC6501028

[39] Park H-K, Hu S, Kim SY, Yoon S, Yoon NG, Lee JH, et al. Pseudohypoxic stabilization of HIF1α via cyclophilin D suppression promotes melanoma metastasis. Signal Transduct Target Ther. 2025;10(1):231. doi: 10.1038/s41392-025-02314-8 40701956 PMC12287346

[40] Guzy RD, Hoyos B, Robin E, Chen H, Liu L, Mansfield KD, et al. Mitochondrial complex III is required for hypoxia-induced ROS production and cellular oxygen sensing. Cell Metab. 2005;1(6):401–8. doi: 10.1016/j.cmet.2005.05.001 16054089

[41] Piantadosi CA, Suliman HB. Transcriptional Regulation of SDHa flavoprotein by nuclear respiratory factor-1 prevents pseudo-hypoxia in aerobic cardiac cells. J Biol Chem. 2008;283(16):10967–77. doi: 10.1074/jbc.M709741200 18252725 PMC2447662

[42] Hayashi Y, Zhang Y, Yokota A, Yan X, Liu J, Choi K, et al. Pathobiological Pseudohypoxia as a Putative Mechanism Underlying Myelodysplastic Syndromes. Cancer Discov. 2018;8(11):1438–57. doi: 10.1158/2159-8290.CD-17-1203 30139811 PMC8783373

[43] Campbell BCV, Khatri P. Stroke. Lancet. 2020;396(10244):129–42. doi: 10.1016/S0140-6736(20)31179-X 32653056

[44] Chamorro Á, Dirnagl U, Urra X, Planas AM. Neuroprotection in acute stroke: targeting excitotoxicity, oxidative and nitrosative stress, and inflammation. Lancet Neurol. 2016;15(8):869–81. doi: 10.1016/S1474-4422(16)00114-9 27180033

[45] Zhou Y, Wang Y, Wang J, Anne Stetler R, Yang Q-W. Inflammation in intracerebral hemorrhage: from mechanisms to clinical translation. Prog Neurobiol. 2014;115:25–44. doi: 10.1016/j.pneurobio.2013.11.003 24291544

[46] Pediaditakis I, Kodella KR, Manatakis DV, Le CY, Barthakur S, Sorets A, et al. A microengineered Brain-Chip to model neuroinflammation in humans. iScience. 2022;25(8):104813. doi: 10.1016/j.isci.2022.104813 35982785 PMC9379671

[47] Chim SM, Howell K, Kokkosis A, Zambrowicz B, Karalis K, Pavlopoulos E. A Human Brain-Chip for Modeling Brain Pathologies and Screening Blood-Brain Barrier Crossing Therapeutic Strategies. Pharmaceutics. 2024;16(10):1314. doi: 10.3390/pharmaceutics16101314 39458643 PMC11510380

[48] Vatine GD, Barrile R, Workman MJ, Sances S, Barriga BK, Rahnama M, et al. Human iPSC-Derived Blood-Brain Barrier Chips Enable Disease Modeling and Personalized Medicine Applications. Cell Stem Cell. 2019;24(6):995-1005.e6. doi: 10.1016/j.stem.2019.05.011 31173718

[49] Kawakita S, Mandal K, Mou L, Mecwan MM, Zhu Y, Li S. Organ-on-a-chip models of the blood-brain barrier: recent advances and future prospects. Small. 2022;18(39):e2201401. doi: 10.1002/smll.202201401 35978444 PMC9529899

[50] Daneman R, Prat A. The blood-brain barrier. Cold Spring Harb Perspect Biol. 2015;7(1):a020412. doi: 10.1101/cshperspect.a020412 25561720 PMC4292164

[51] Greene C, Hanley N, Campbell M. Blood-brain barrier associated tight junction disruption is a hallmark feature of major psychiatric disorders. Transl Psychiatry. 2020;10(1):373. doi: 10.1038/s41398-020-01054-3 33139732 PMC7606459

[52] Napoli I, Neumann H. Microglial clearance function in health and disease. Neuroscience. 2009;158(3):1030–8. doi: 10.1016/j.neuroscience.2008.06.046 18644426

[53] Holzreuter MA, Segerink LI. Innovative electrode and chip designs for transendothelial electrical resistance measurements in organs-on-chips. Lab Chip. 2024;24(5):1121–34. doi: 10.1039/d3lc00901g 38165817 PMC10898416

[54] Srinivasan B, Kolli AR, Esch MB, Abaci HE, Shuler ML, Hickman JJ. TEER measurement techniques for in vitro barrier model systems. J Lab Autom. 2015;20(2):107–26. doi: 10.1177/2211068214561025 25586998 PMC4652793

