# Supplementary material for: A human 3D BBB chip model of acute stroke simulating a reversible penumbra
Source: PLoS One. 2026 Jul 14;21(7):e0352263. doi: 10.1371/journal.pone.0352263 (PMC13367901; doi:10.1371/journal.pone.0352263)
Supplement: Supplementary Materials 1 — (DOCX) [file pone.0352263.s001.docx]

**Supplementary Results**

**Cell Culture and Stroke Induction**

Preliminary 2D culture experiments were conducted to refine the stroke and treatment protocol for the Transwell/organ-on-chip BBB model. The results below correspond to S1 Fig. which summarizes the findings from these optimization tests. Schematic workflow illustrating the development and validation of in vitro stroke and hypothermia models using 2D and 3D blood–brain barrier (BBB) platforms. In the first phase, a 2D monolayer culture system was optimized to determine the appropriate conditions for ischemic injury and hypothermia-based recovery. Human brain microvascular endothelial cells (HBMECs), astrocytes, and pericytes were individually exposed to antimycin A to simulate stroke, followed by hypothermic treatment to assess cell-type specific responses. Based on these results, two types of 3D BBB models—a microfluidic organ-on-chip system (Emulate Brain-Chip) and a conventional Transwell co-culture—were constructed using the same cell types to replicate the neurovascular unit. Functional validation of the models was performed via immunocytochemistry (ICC) for tight junction proteins, transendothelial electrical resistance (TEER) measurement, and FITC-dextran permeability assays.

**Stroke Induction in 2D Monocultures**

Treatment with antimycin A for 1 hour caused a significant decrease in cell viability across all three cell types. HBMECs, astrocytes, and pericytes each showed markedly reduced viable cell fractions compared to untreated controls, confirming effective induction of ischemic injury. This 1-hour chemical ischemia condition (25 μM antimycin A) was therefore selected as the optimal stroke induction, as shorter exposures caused minimal cell death while longer exposures did not further reduce viability (S2, S3 Fig.).

**Endothelial Tight Junction Disruption**

We next confirmed that the stroke-mimicking insult compromises endothelial barrier properties. Immunofluorescence staining of tight junction proteins in HBMECs revealed clear differences between control and stroke conditions (S4 A Fig.). In normal (untreated) endothelial cultures, ZO-1 and claudin-5 were continuously localized at cell–cell borders, indicating intact cell junctions. In contrast, endothelial cells fixed immediately after the 1-hour antimycin A treatment showed discontinuous and patchy ZO-1/claudin-5 staining (S4 B Fig.). This loss of continuous junctional signal confirms that our *in vitro* stroke conditions disrupt endothelial tight junction integrity, mimicking blood–brain barrier (BBB) breakdown.

**Cell Recovery During Reperfusion**

The ability of cells to recover after ischemic injury was assessed by reintroducing normal culture conditions for 48 hours post-insult. Notably, HBMEC cultures that had undergone the 1-hour stroke induction were able to re-form a confluent monolayer after 48 hours of reperfusion. Phase-contrast microscopy at 48 hours showed that previously injured endothelial cells spread and regained cell–cell contacts, although the monolayer remained slightly less uniform compared to uninjured controls (S5 Fig.). This indicates partial recovery of the endothelial layer with extended reperfusion, supporting the inclusion of a prolonged recovery phase in the protocol.

**Hypothermia Treatment Efficacy**

Mild hypothermia during reperfusion had a visibly protective effect on cells following stroke injury. When cultures were maintained at 33 °C for the 48-hour reperfusion period, cell morphology and coverage were improved relative to the normothermic reperfusion condition. Endothelial cells in the hypothermia group appeared more polygonal and tightly packed, similar to uninjured controls, whereas cells at 37 °C showed areas of cell thinning. Moreover, qualitative viability appeared higher with 33 °C treatment, suggesting reduced delayed cell death. These observations validated 33 °C as an effective therapeutic hypothermia condition, which was incorporated into the final protocol (S6 Fig.).

**Cell Viability Assays in the Emulate Organ-on-Chip Model**

The cytotoxic response of the Emulate organ-on-chip BBB model to antimycin A–induced ischemic injury was assessed using a Calcein-AM/PI live/dead assay. Endothelial cell viability decreased in a dose-dependent manner with increasing concentrations of antimycin A. Treatment with 1.25 μM resulted in 96.1% viability, while 2.5 μM, 5 μM, and 10 μM led to reduced viability levels of 68.6%, 43.2%, and 24.9%, respectively. These findings indicate a sharp decline in viability at concentrations above 2.5 μM, suggesting that 2.5 μM represents a threshold dose for partially reversible ischemic damage in the BBB chip model. (S7 Fig.).

**Final Optimized Protocol**

Based on the above findings, we established a standardized in vitro stroke–reperfusion experiment with three comparative conditions (summarized in S1 Table). In all groups, ischemic injury is induced by a 1-hour antimycin A treatment. (1) Stroke only: Cells receive the 1-hour injury and are then immediately processed for endpoints (no recovery period). (2) Stroke + Reperfusion: After the 1-hour injury, cells are returned to 37 °C with fresh medium for an additional 47 hours of incubation (normothermic reperfusion) before analysis. (3) Stroke + Reperfusion + Hypothermia: After the 1-hour injury, cells are kept at 33 °C in fresh medium for 47 hours (hypothermic reperfusion) before analysis. The total experimental duration for conditions (2) and (3) is 48 hours (including the initial 1-hour ischemia). This optimized protocol allows comparison of acute ischemic damage versus post-ischemic recovery, as well as evaluation of mild hypothermia as an intervention during reperfusion.


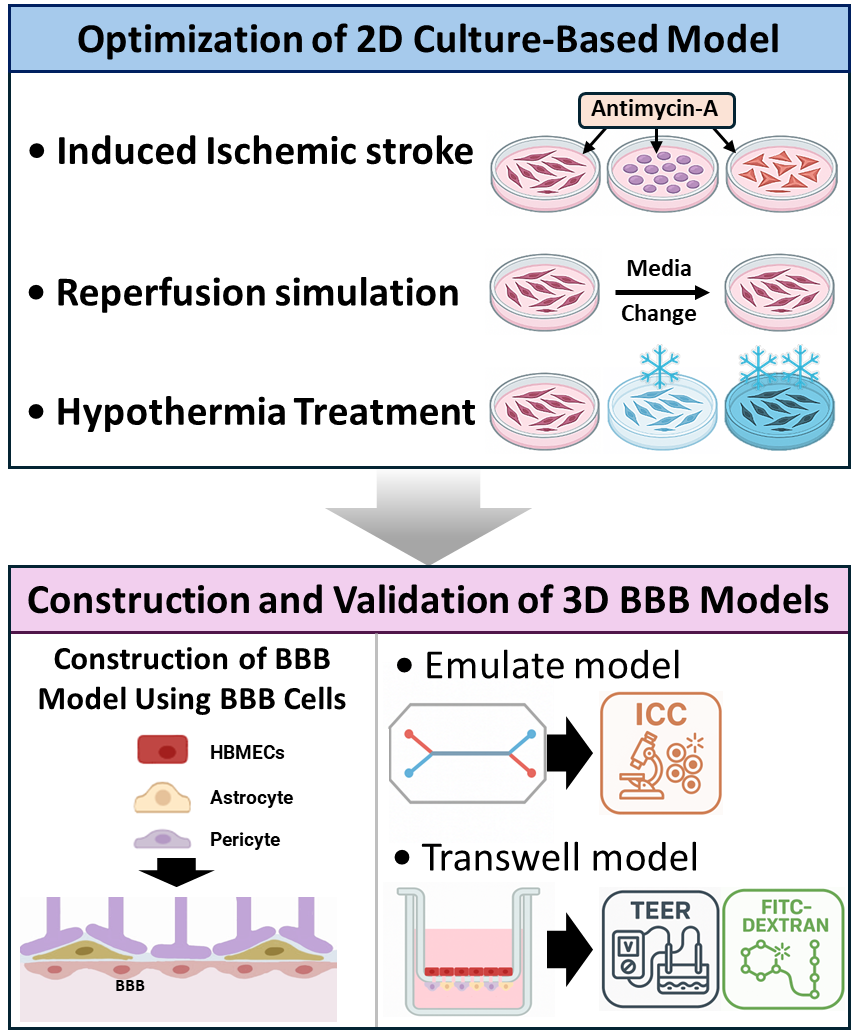


**S1 Fig. Optimization of 2D Culture-Based Model: Acute ischemic conditions were induced in a 2D culture by antimycin A.** Reperfusion was simulated by replacing the medium, and therapeutic hypothermia was applied via temperature reduction to 33°C. These steps were optimized to evaluate cellular responses under stroke-relevant conditions. Construction and Validation of 3D BBB Models: Triculture BBB models incorporating HBMECs, astrocytes, and pericytes were developed using Transwell inserts and Emulate chips. Barrier integrity and function were validated using immunocytochemistry (ICC), transendothelial electrical resistance (TEER), and FITC-dextran permeability assays.


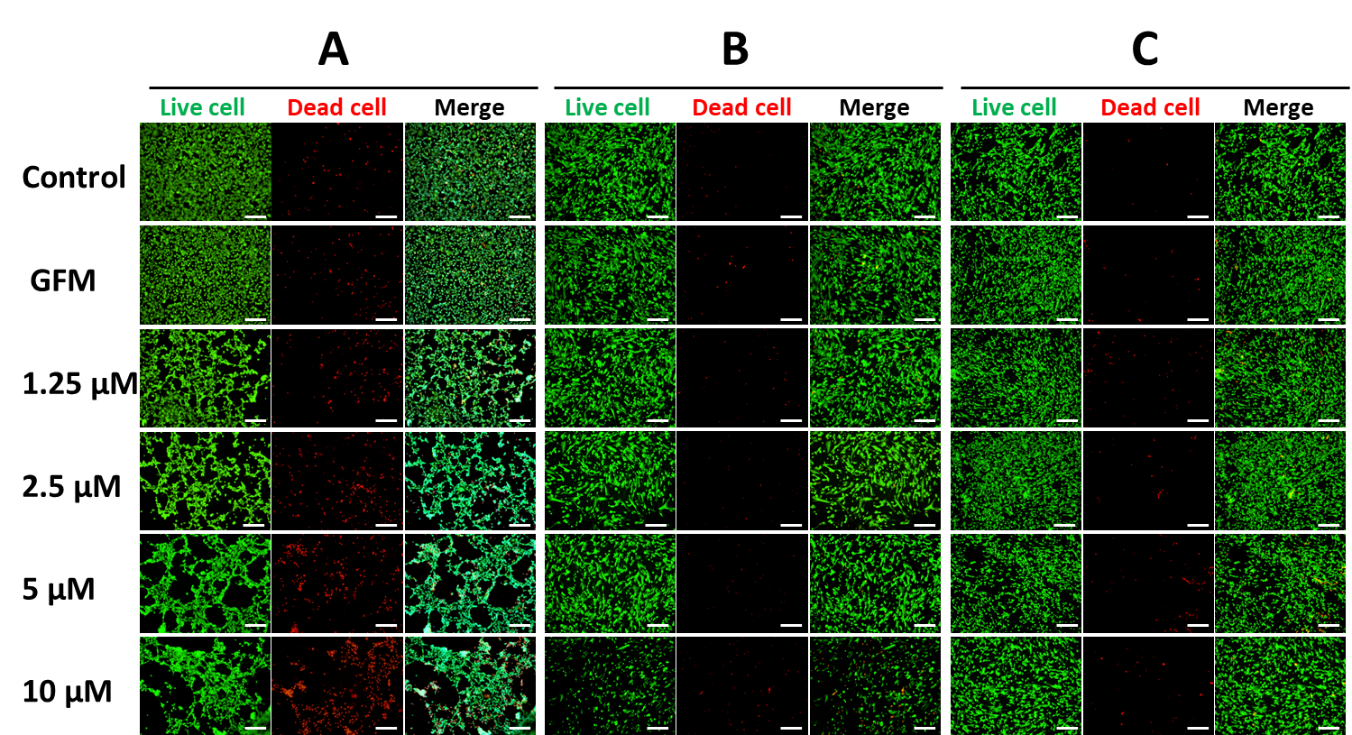


**S2 Fig. Cell viability assay following antimycin A induced ischemic injury in 2D culture.** HBMECs (A), astrocytes (B), and pericytes (C) were cultured under standard medium, glucose-free medium (GFM), or GFM supplemented with 1.25, 2.5, 5, or 10 μM antimycin A for 1 h or 3 h. Cells were stained with Calcein-AM (green; live) and propidium iodide (PI; red; dead). Representative fluorescence images from the 1 hour treatment groups are shown, and corresponding viability quantification is presented in Fig. 3. Data are derived from independent experiments independent experiments (n=4). Scale bar = 200 μm


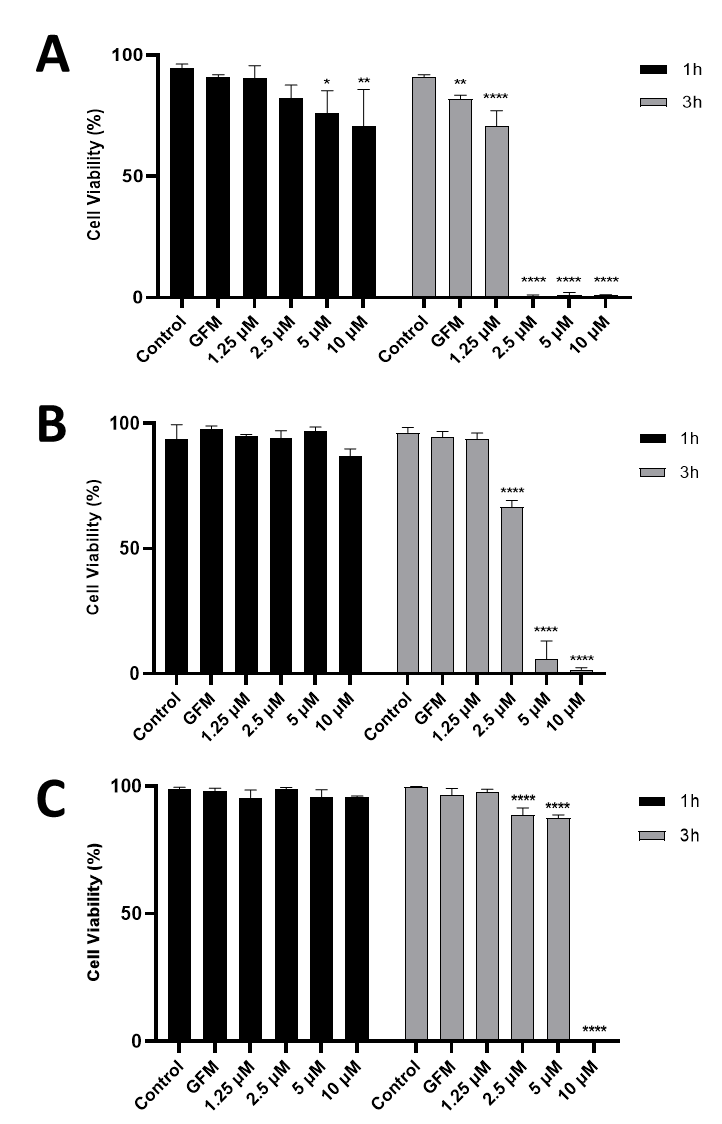


**S3 Fig. Quantitative analysis of cell viability assay after ischemic injury.** Bar graphs show the percentage of viable HBMECs (A), astrocytes (B), and pericytes (C) after 1-hour exposure to antimycin A (1.25–10 μM), based on Calcein-AM–positive cell ratios. Data are presented as mean ± SE (n = 4 independent experiments); statistical significance was assessed by one-way ANOVA with Tukey’s post-hoc test (^*^P < 0.05, ^**^P < 0.01, ^***^P < 0.001, ^****^P < 0.0001).


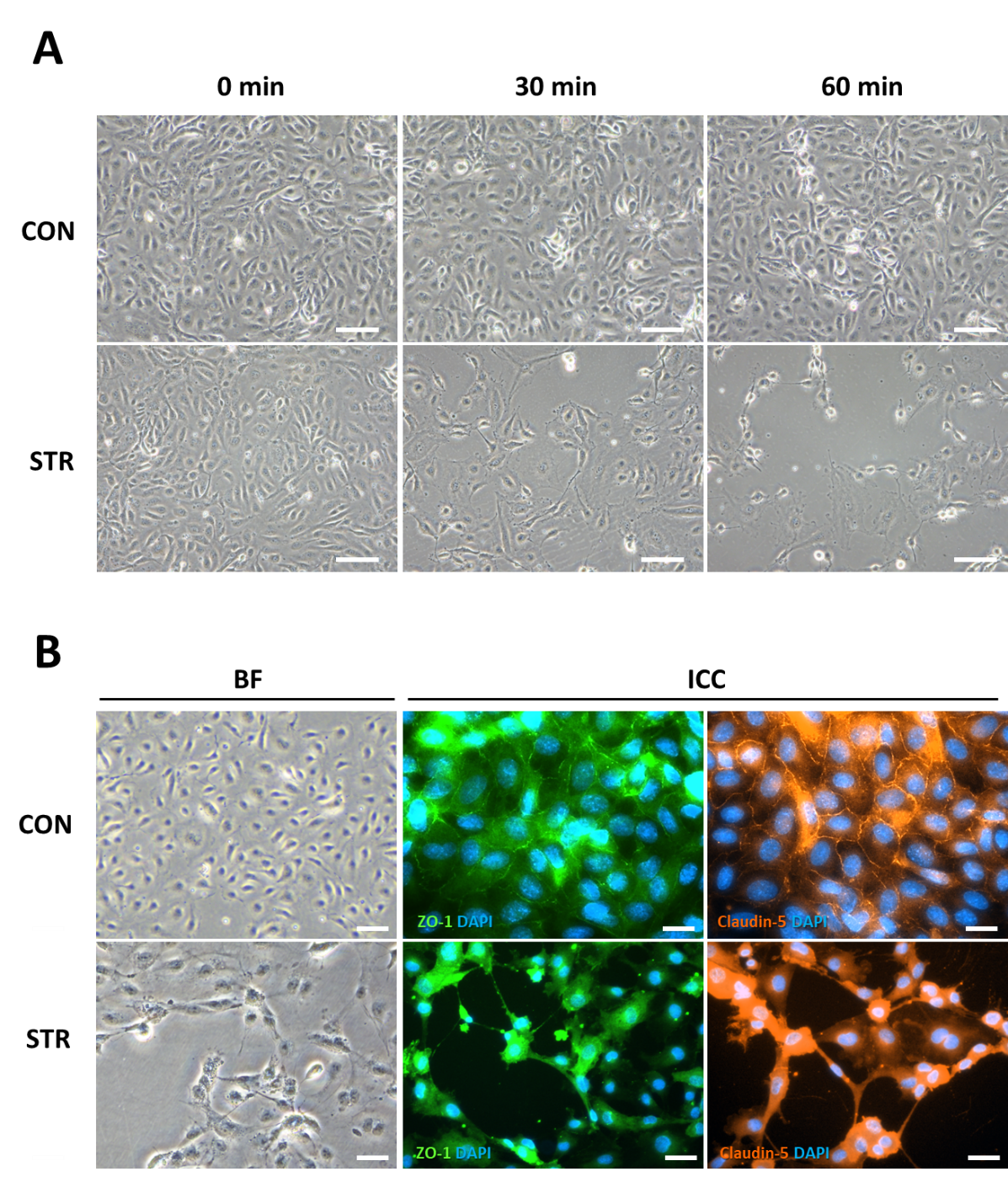


**S4 Fig. Morphological alterations and disruption of tight junctions in 2D cultured HBMECs under antimycin A induced ischemic conditions.** (A) Brightfield images show morphological changes in HBMECs following exposure to 2.5 μM antimycin A for 30 or 60 minutes. Control cells exhibited flat, polygonal or fusiform morphology forming a confluent monolayer. After 60 minutes of treatment, cells displayed a star-shaped appearance, with intercellular gaps and disrupted monolayer integrity. (Scale bar = 100 μm.). (B) Immunofluorescence images of tight junction proteins ZO-1 (green) and claudin-5 (orange) following 1-hour exposure to antimycin A (2.5 μM). In controls, both proteins were continuously localized at cell borders. Post-treatment, staining appeared fragmented and discontinuous, indicating tight junction disruption. Nuclei were stained with DAPI (blue) (Scale bar = 50 μm). Images are representative of independent experiments (n = 3).


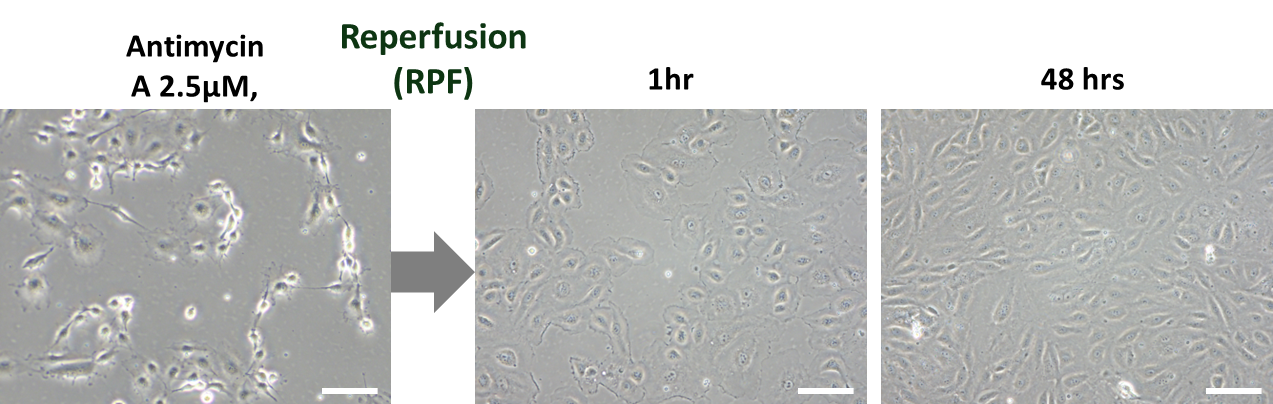


**S5 Fig. Morphological changes in HBMECs after antimycin A–induced ischemia and reperfusion in 2D culture.** Phase-contrast images show HBMECs treated with 2.5 μM antimycin A for 1 hour to induce ischemia, followed by medium replacement simulating reperfusion (RPF). Morphological assessment at 1-hour post-RPF revealed partial monolayer disruption and widened intercellular gaps, which markedly recovered by 48 hours. Scale bar = 100 μm. Images are representative of independent experiments (n = 3).


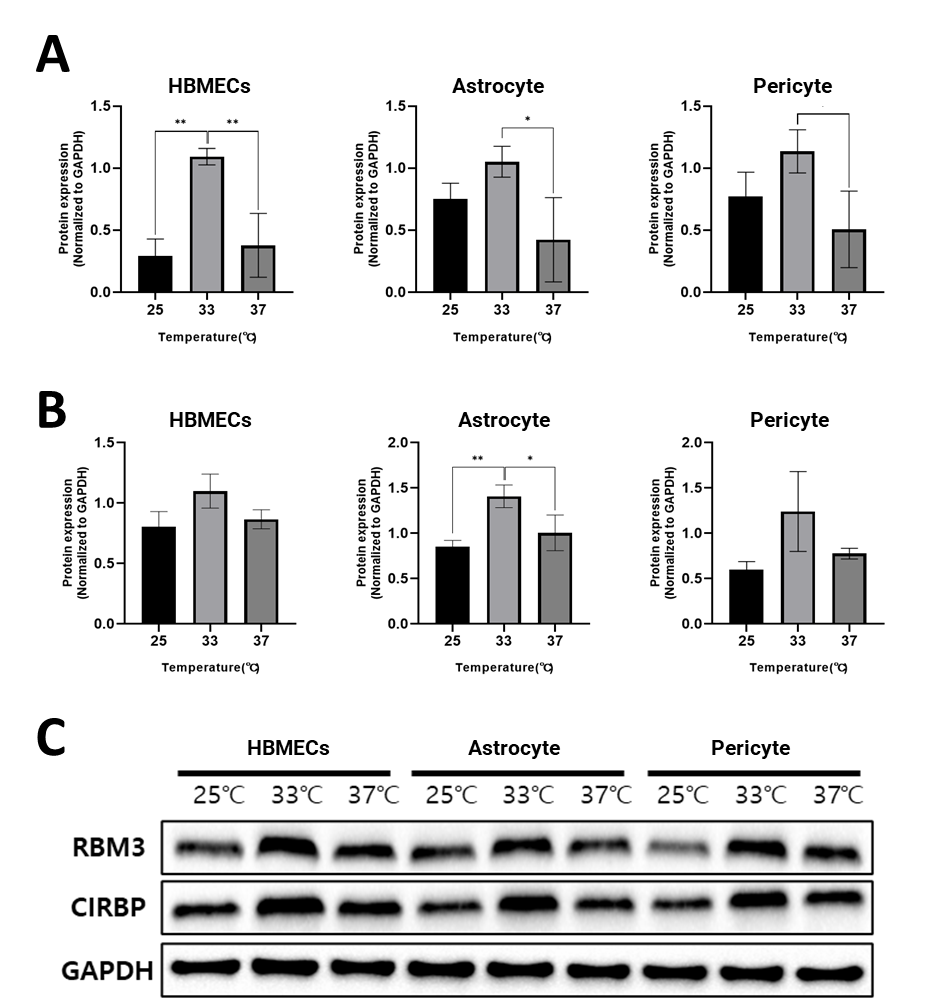


**S6 Fig. Western blot analysis of RBM3 and CIRBP expression in 2D-cultured HBMECs, astrocytes, and pericytes under hypothermic conditions.** Cells were exposed to 31°C, 33°C, or 35°C to determine the optimal temperature for hypothermia treatment. (A, B) Bar graphs show quantified RBM3 and CIRBP expression levels, respectively (mean ± SD, n = 3 per group). (C) Representative Western blots of RBM3 (17 kDa), CIRBP (17 kDa), and GAPDH (40 kDa; loading control), corresponding to the quantified data (n = 3 per group). RBM3 expression peaked at 33°C in all cell types with statistical significance, while CIRBP also showed maximal expression at 33°C—significant in astrocytes and trending higher in HBMECs and pericytes. Statistical analysis was conducted using one-way ANOVA with Tukey’s post hoc test; *p < 0.05, **p < 0.01.


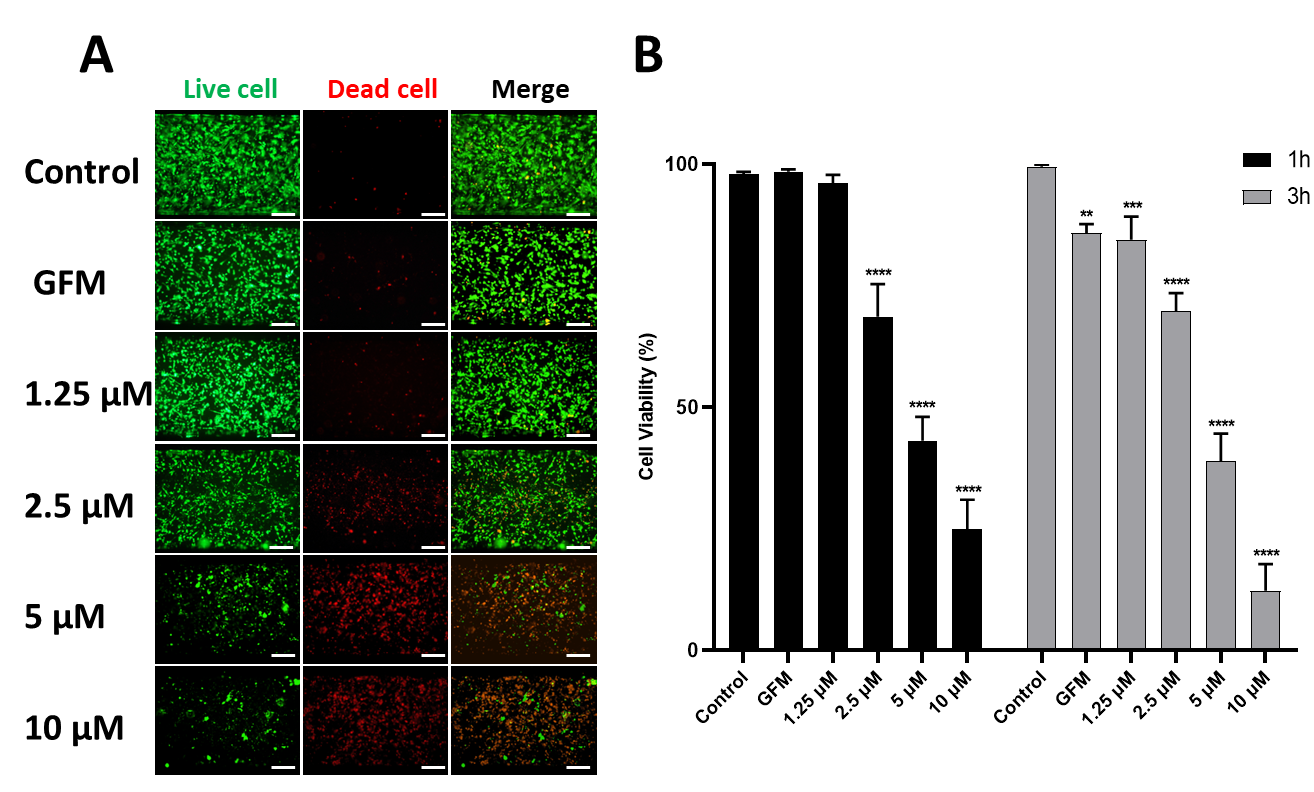


**S7 Fig. Cell viability assessment in the Emulate BBB chip following acute ischemic stroke induction.** (A) Representative fluorescence images of HBMECs in the Emulate BBB chip after 1-hour ischemic stroke induction using glucose-free medium (GFM) alone or GFM supplemented with increasing concentrations of antimycin A (1.25, 2.5, 5, or 10 μM). Standard culture medium was used as the control. Although both 1-hour and 3-hour treatments were performed, only 1-hour images are shown. Live and dead cells were stained with calcein-AM (green) and propidium iodide (PI, red), respectively. Scale bar = 100 μm. Images are representative of independent experiments (n = 4).
(B) Quantification of cell viability based on calcein-AM/PI staining. Data are presented as mean ± standard error (SE) from four independent experiments (n = 4 per condition). Statistical analysis was performed using one-way ANOVA followed by Tukey’s post hoc test; *P < 0.05, **P < 0.01, ***P < 0.001, ****P < 0.0001.

**
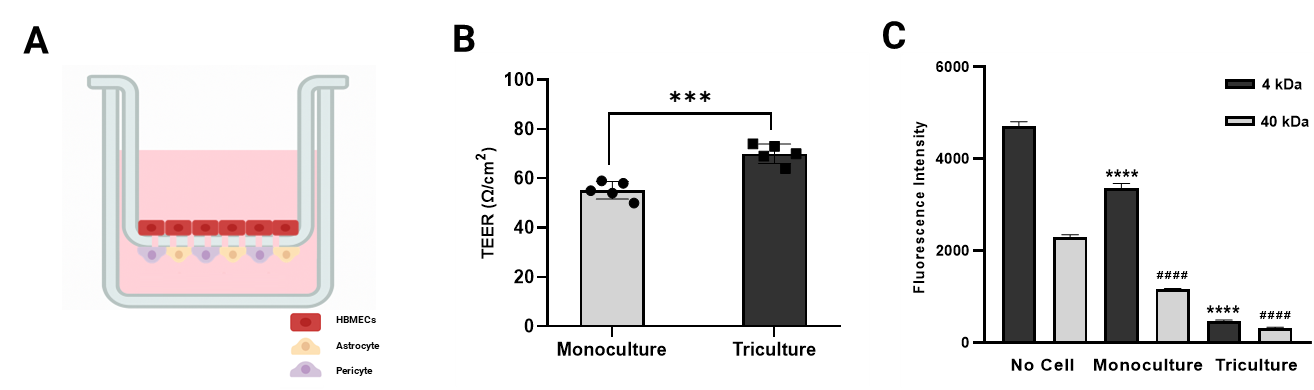
**

**S8** **Fig. Barrier characterization of the Transwell model.** (A) Schematic diagram of the Transwell triculture setup (HBMECs on the upper surface of the insert membrane; astrocytes and pericytes on the underside of the membrane).  (B) TEER measurements comparing HBMEC monoculture vs. the triculture model. The triculture exhibits significantly higher resistance than the monoculture (n = 3 per group). (C) FITC-dextran permeability assay showing reduced flux of 4 kDa and 40 kDa tracers in the triculture compared to controls. Data are presented as mean ± SE (n = 3 per group).. Statistical comparisons were made by unpaired t-tests; **P < 0.0001 vs. no-cell control, ^####^P < 0.0001 vs. HBMEC monoculture.

**S1 Table. Final experimental conditions for the in vitro ischemia–reperfusion injury model (total duration 48 h)**

| **Condition** | **Ischemic Insult (1 h)** | **Post-insult Treatment** | **Reperfusion Temperature** |
| --- | --- | --- | --- |
| **Stroke only** | 1 h antimycin A (2.5 μM) | None (no reperfusion period) | N/A (no reperfusion) |
| **Stroke + Reperfusion** | 1 h antimycin A (2.5 μM) | 48 h recovery in fresh medium (normoxia) | 37 °C (normothermia) |
| **Stroke + Reperfusion + Hypothermia** | 1 h antimycin A (2.5 μM) | 48 h recovery in fresh medium (normoxia) | 33 °C (mild hypothermia) |

**S9 Dataset. Raw viability data corresponding to S3 Fig A.
S10 Dataset. Raw viability data corresponding to S3 Fig B.
S11 Dataset. Raw viability data corresponding to S3 Fig C.
S12 Dataset. Raw western blot quantification data corresponding to S6 Fig A.
S13 Dataset. Raw western blot quantification data corresponding to S6 Fig B.
S14 File. Uncropped western blot image for RBM3 used in S6 Fig C.
S15 File. Uncropped western blot image for CIRBP used in S6 Fig C.
S16 File. Uncropped western blot image for GAPDH used in S6 Fig C.
S17 Dataset. Raw viability data corresponding to S7 Fig B.
S18 Dataset. Raw FITC-dextran permeability data corresponding to S8 Fig C.
S19 Dataset. Raw TEER measurement data corresponding to S8 Fig B.
S20 File. Uncropped western blot image for RBM3 used in Fig 3A.
S21 File. Uncropped western blot image for GAPDH used in Fig 3A.
S22 File. Uncropped western blot image for CIRBP used in Fig 3A.
S23 File. Uncropped western blot image for HIF-1α used in Fig 3A.
S24 Dataset. Raw data for CIRBP western blot quantification shown in Fig 3A.
S25 Dataset. Raw data for HIF-1α western blot quantification shown in Fig 3A.
S26 Dataset. Raw data for RBM3 western blot quantification shown in Fig 3A.
S27 Dataset. Raw data for real-time PCR analysis shown in Fig 3C.**
