# Supplementary figures and images for: A human 3D BBB chip model of acute stroke simulating a reversible penumbra
Source: PLoS One. 2026 Jul 14;21(7):e0352263. doi: 10.1371/journal.pone.0352263 (PMC13367901; doi:10.1371/journal.pone.0352263)

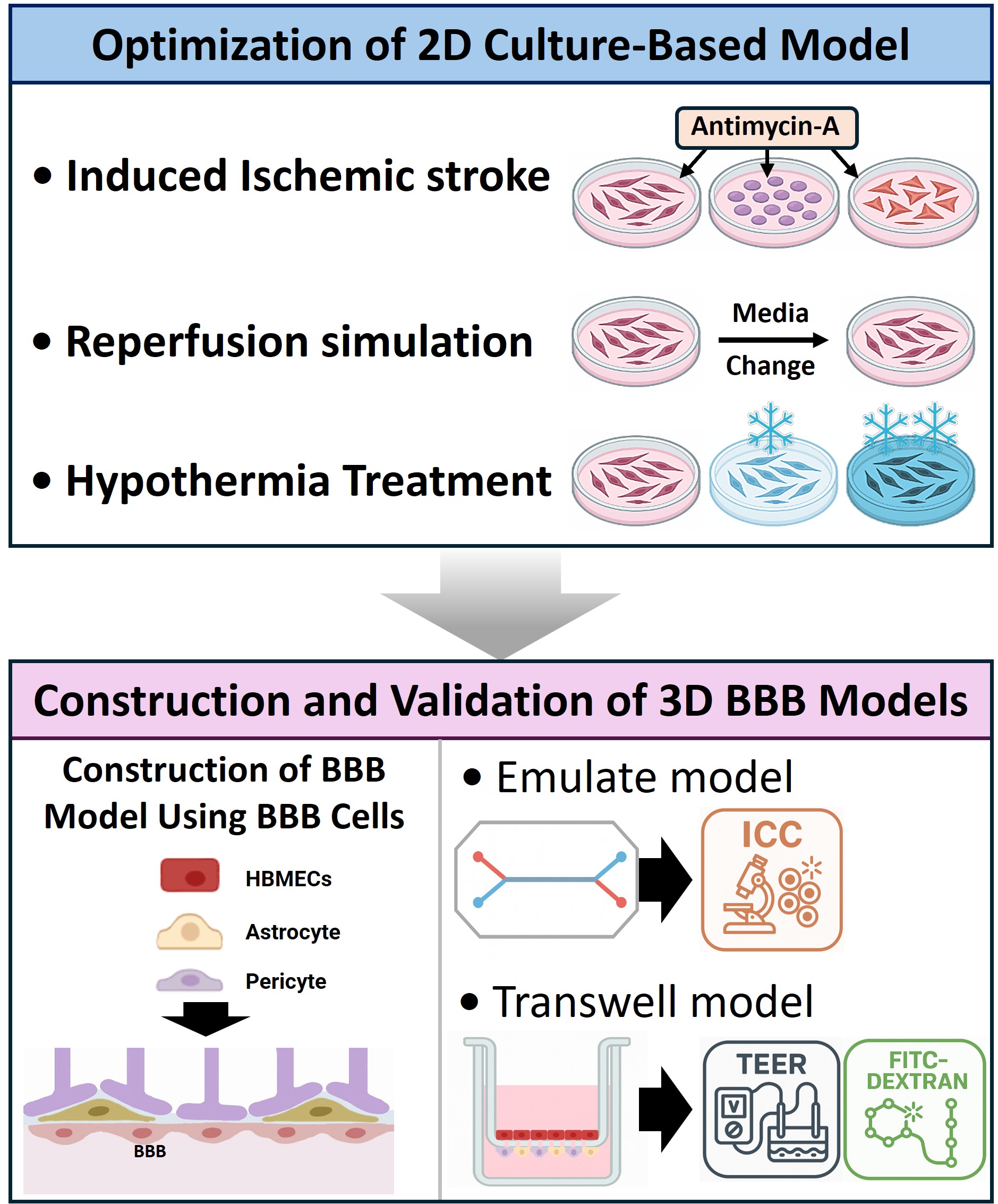

Supplement: S1 Fig — (JPG) [file pone.0352263.s002.jpg]

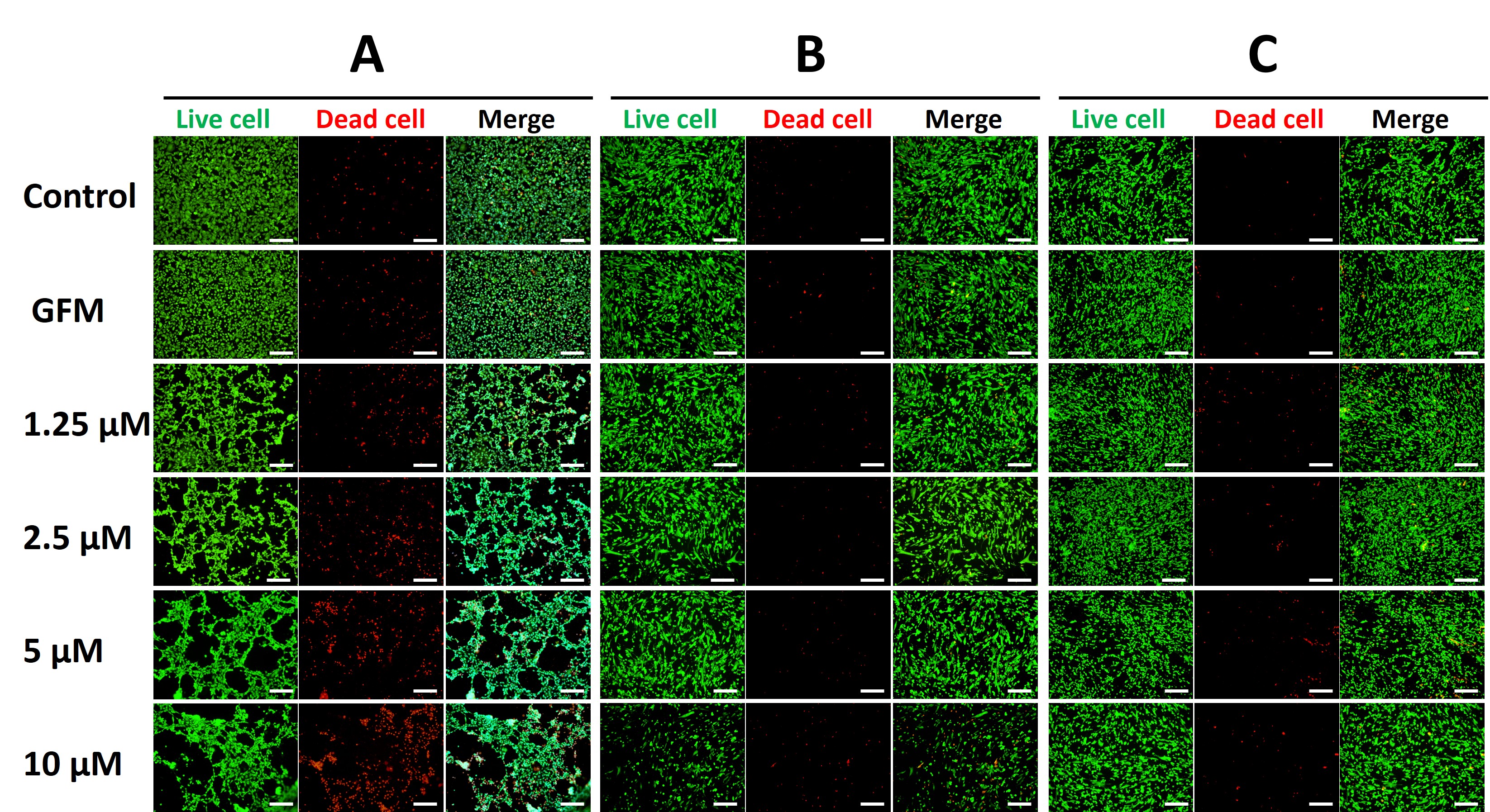

Supplement: S2 Fig — (JPG) [file pone.0352263.s003.jpg]

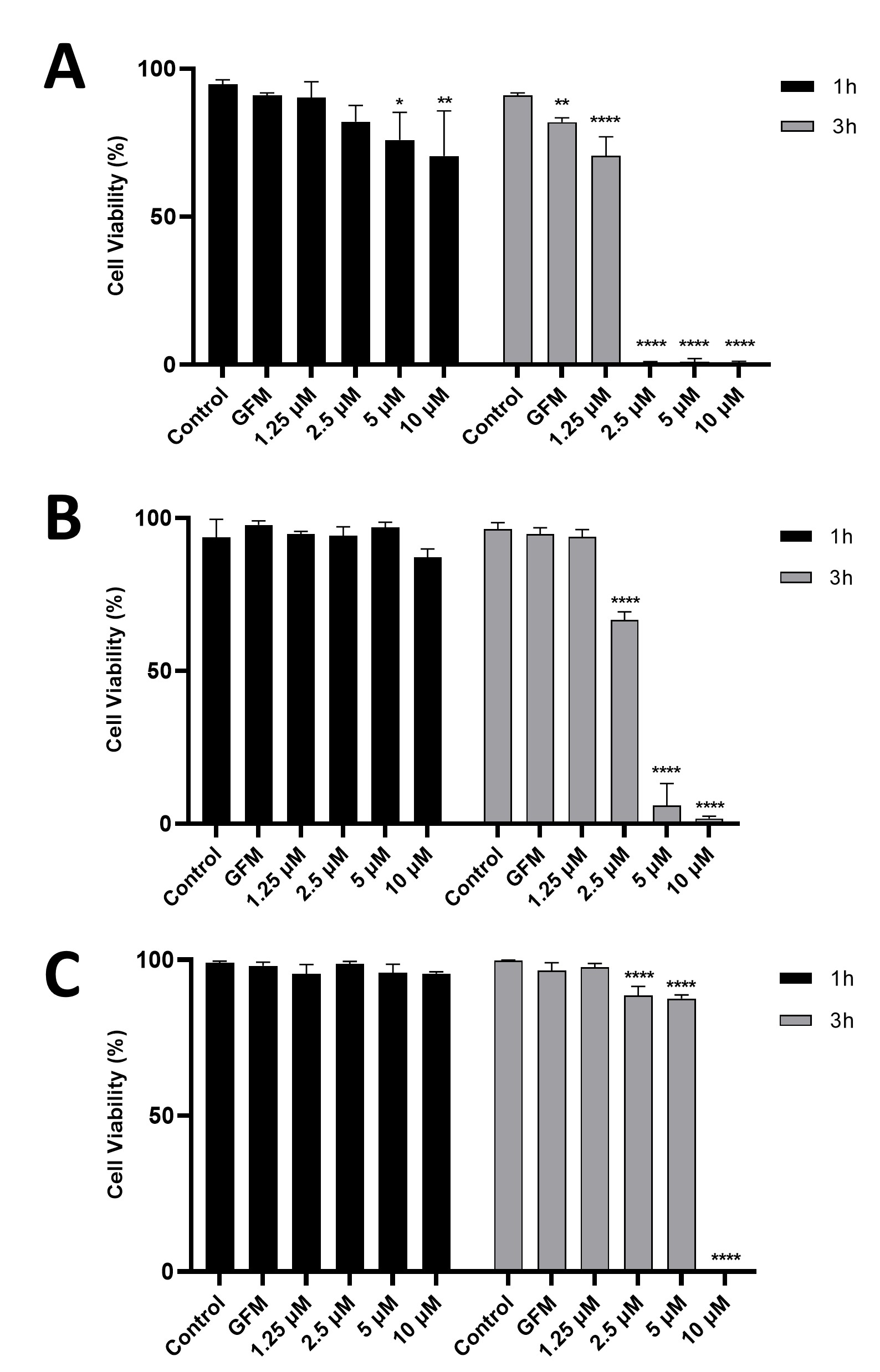

Supplement: S3 Fig — (JPG) [file pone.0352263.s004.jpg]

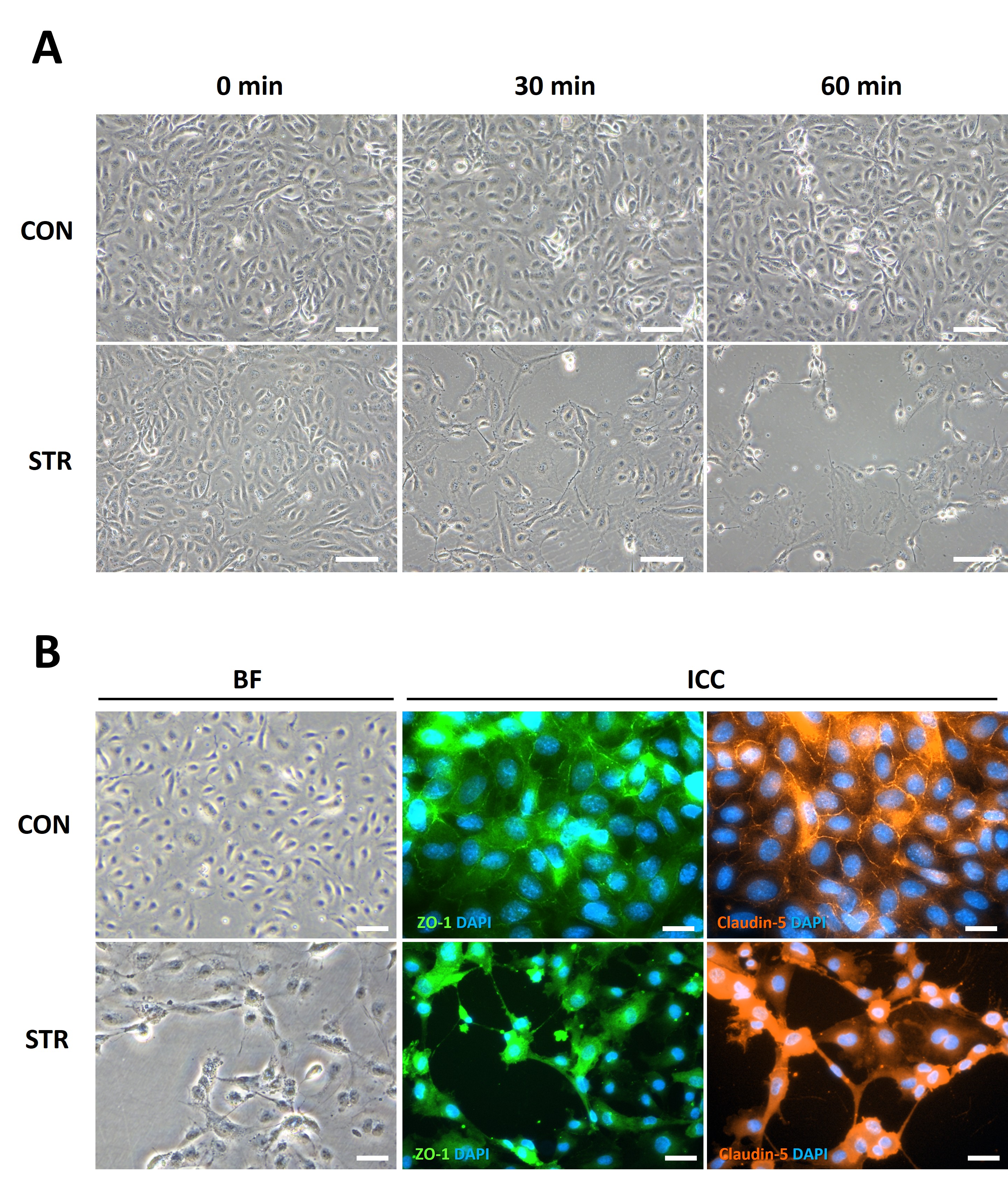

Supplement: S4 Fig — (JPG) [file pone.0352263.s005.jpg]

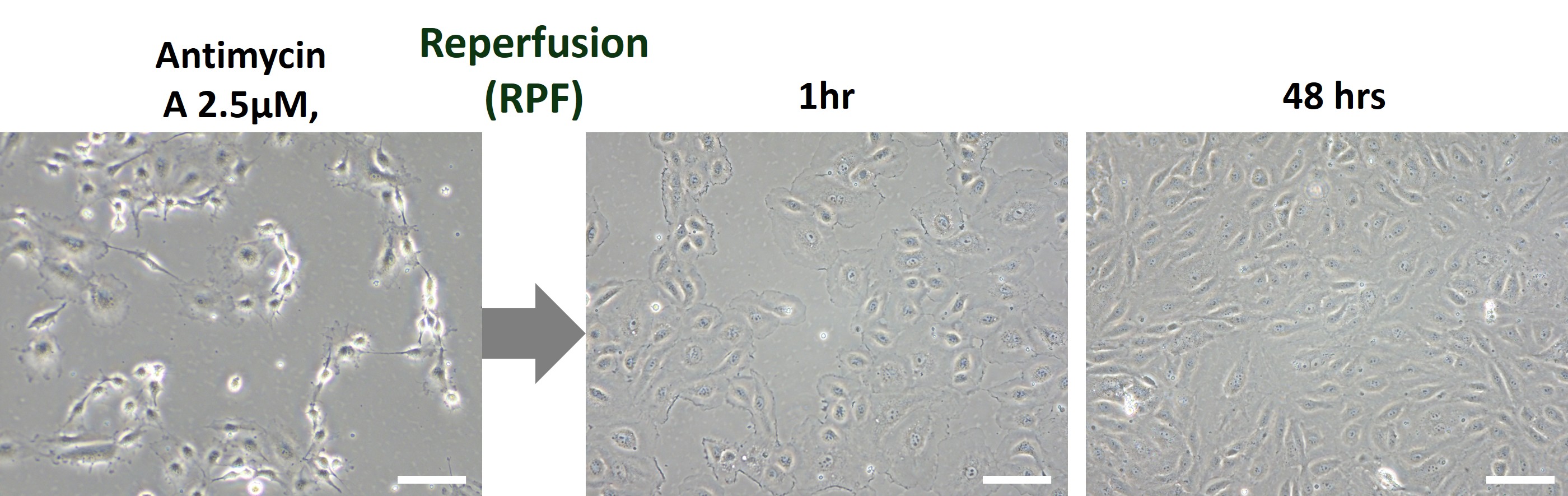

Supplement: S5 Fig — (JPG) [file pone.0352263.s006.jpg]

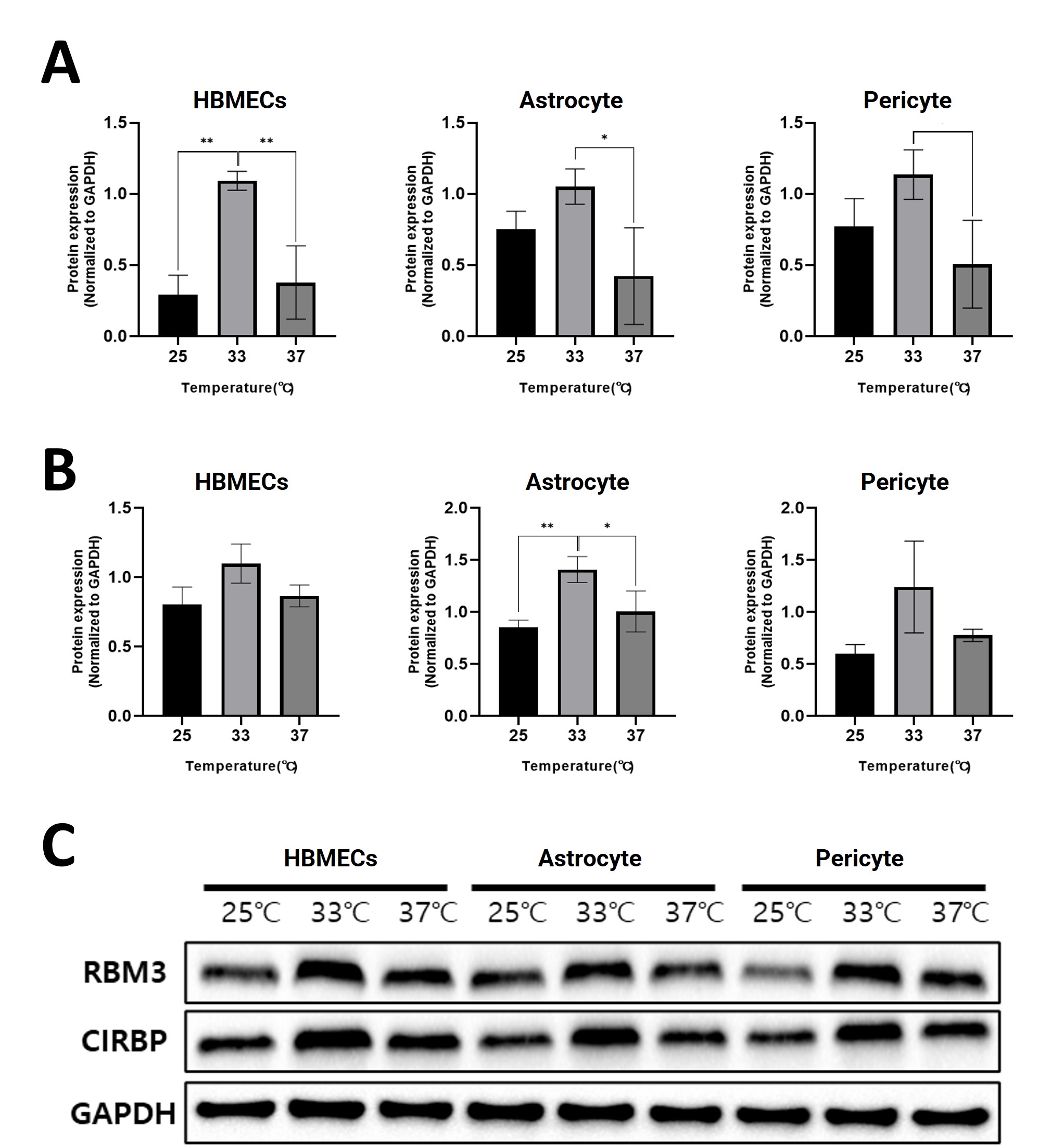

Supplement: S6 Fig — (JPG) [file pone.0352263.s007.jpg]

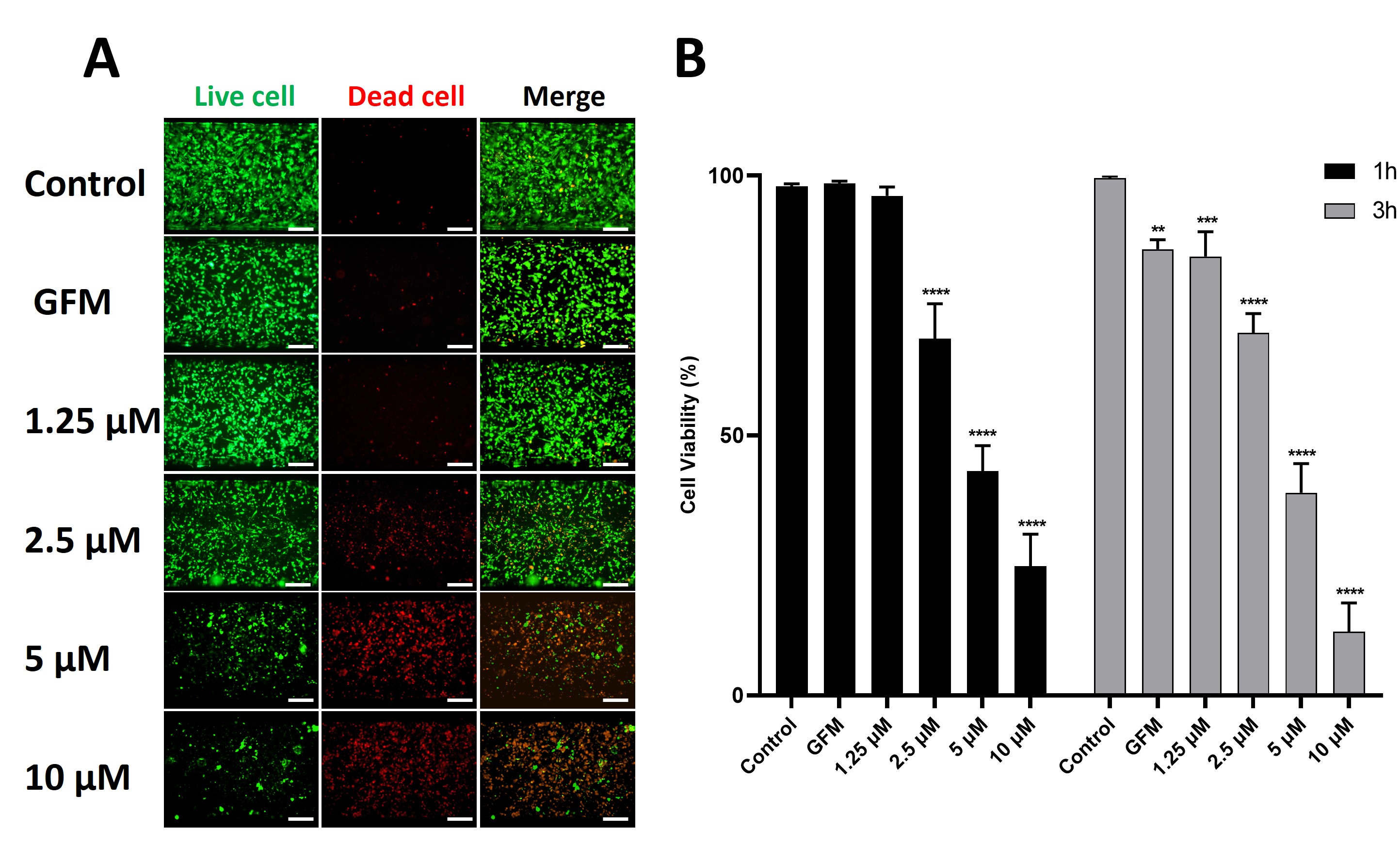

Supplement: S7 Fig — (JPG) [file pone.0352263.s008.jpg]

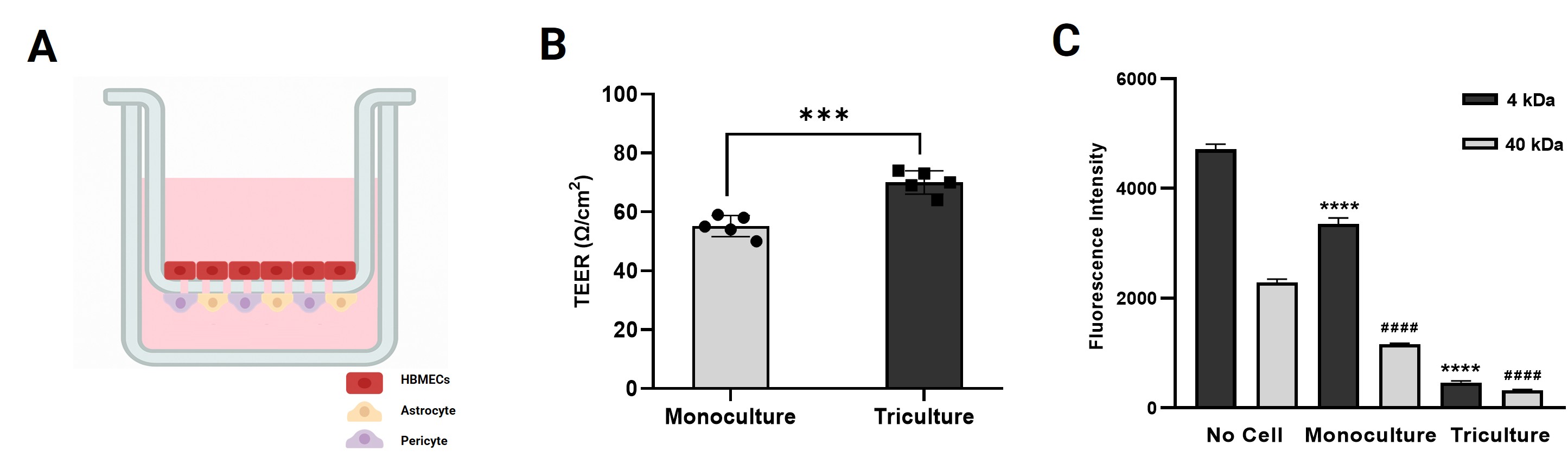

Supplement: S8 Fig — (JPG) [file pone.0352263.s009.jpg]

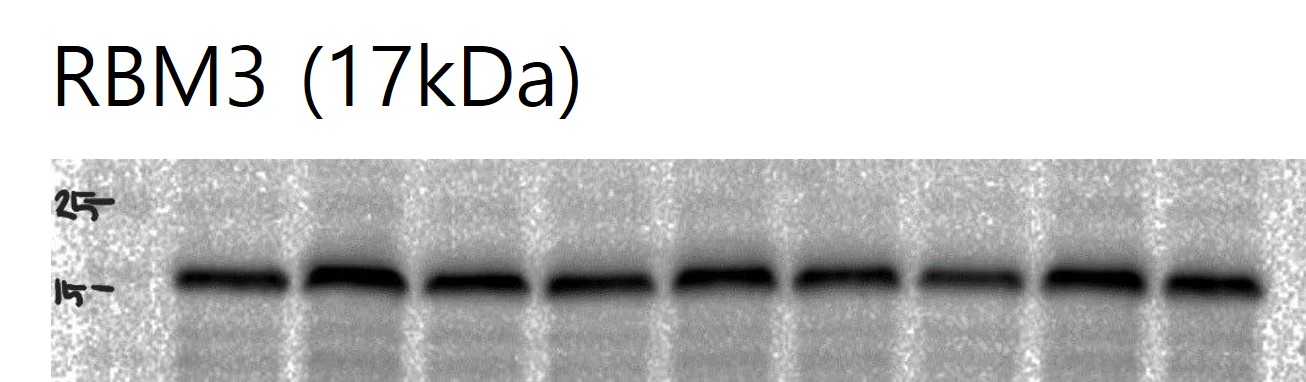

Supplement: S13 File — (JPG) [file pone.0352263.s023.jpg]

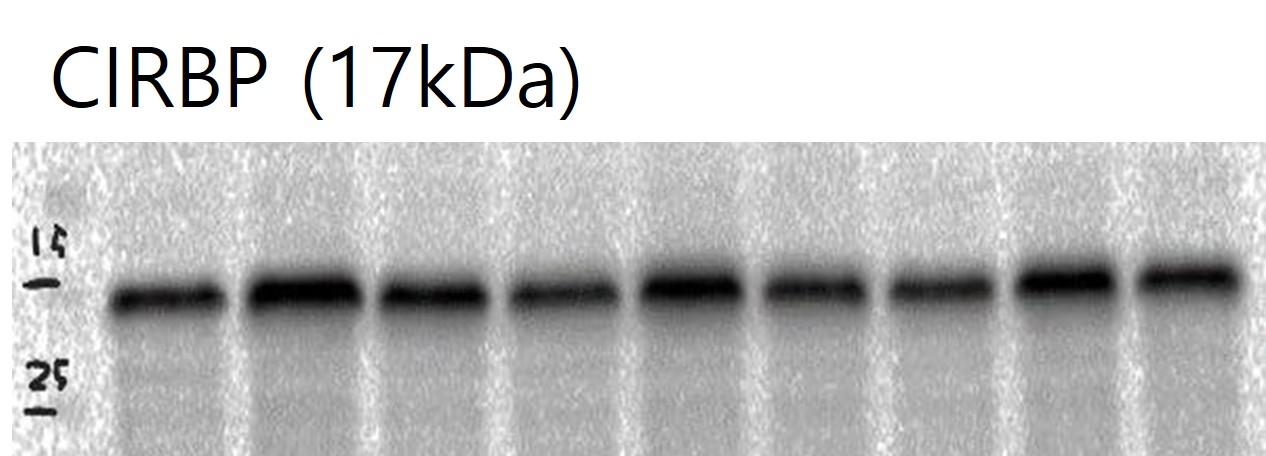

Supplement: S14 File — (JPG) [file pone.0352263.s024.jpg]

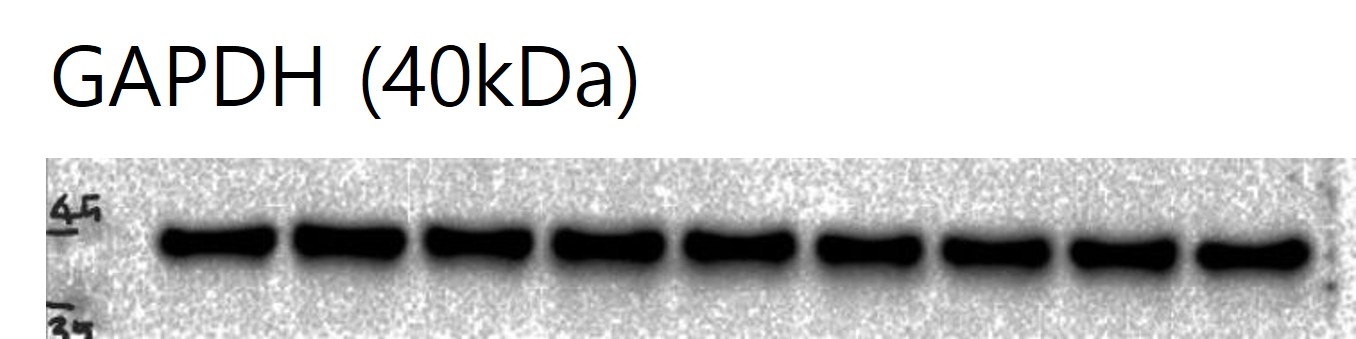

Supplement: S1 File — (JPG) [file pone.0352263.s025.jpg]

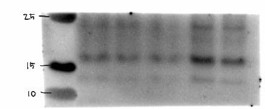

Supplement: S2 File — (TIFF) [file pone.0352263.s026.tiff]

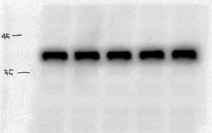

Supplement: S3 File — (TIFF) [file pone.0352263.s027.tiff]

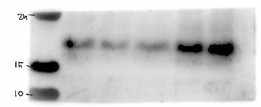

Supplement: S4 File — (TIFF) [file pone.0352263.s028.tiff]

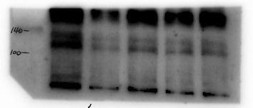

Supplement: S5 File — (TIFF) [file pone.0352263.s029.tiff]
