# Supplementary material for: A human 3D BBB chip model of acute stroke simulating a reversible penumbra
Source: PLoS One. 2026 Jul 14;21(7):e0352263. doi: 10.1371/journal.pone.0352263 (PMC13367901; doi:10.1371/journal.pone.0352263)
Supplement: S1 Table — (PDF) [file pone.0352263.s010.pdf]

S1 Table. Final experimental conditions for the in vitro ischemia–reperfusion injury model (total duration 48 h)

Condition	Ischemic Insult (1 h)	Post-insult Treatment	Reperfusion Temperature
**Stroke only**	1 h antimycin A (2.5 μM)	None (no reperfusion period)	N/A (no reperfusion)
**Stroke + Reperfusion**	1 h antimycin A (2.5 μM)	48 h recovery in fresh medium (normoxia)	37 °C (normothermia)
**Stroke + Reperfusion + Hypothermia**	1 h antimycin A (2.5 μM)	48 h recovery in fresh medium (normoxia)	33 °C (mild hypothermia)
